# New Insights into the Epidemiology of Vulvar Cancer: Systematic Literature Review for an Update of Incidence and Risk Factors

**DOI:** 10.3390/cancers14020389

**Published:** 2022-01-13

**Authors:** Lauro Bucchi, Margherita Pizzato, Stefano Rosso, Stefano Ferretti

**Affiliations:** 1 Romagna Cancer Registry, Romagna Cancer Institute, Istituto Romagnolo per lo Studio dei Tumori “Dino Amadori”, IRST, IRCCS, Meldola, 47014 Forlì, Italy; 2 Piedmont Cancer Registry, Centre for Epidemiology and Prevention in Oncology in Piedmont, 10126 Turin, Italy; pizzato.margherita@gmail.com (M.P.); stefano.rosso@cpo.it (S.R.); 3 Department of Clinical Sciences and Community Health, Università degli Studi di Milano, 20122 Milan, Italy; 4 Department of Morphology, Surgery and Experimental Medicine, University of Ferrara, and Local Health Authority, 44121 Ferrara, Italy; stefano.ferretti@unife.it

**Keywords:** vulvar cancer, vulval cancer, epidemiology, incidence, risk factor

## Abstract

**Simple Summary:**

Vulvar cancer incidence data were sought from official sources (WHO *Cancer Incidence in Five Continents*) and studies reporting comparable data. With respect to risk factors, a systematic PubMed search of articles published since 1980 identified 69 original cohort and case-control studies. Information was extracted using a PRISMA predesigned data collection form. Recent advances have provided further evidence in support of the carcinogenic model centred on human papillomavirus infection with different defects of the immune function. Conversely, the model centred on the role of vulvar lichen sclerosus and the often-associated differentiated vulva intraepithelial neoplasia has continued to be understudied.

**Abstract:**

The aim of this review was an update of vulvar cancer incidence rates and trends and of all known and putative risk factors for the disease. The most recent incidence data were sought from official sources (WHO *Cancer Incidence in Five Continents*). To obtain an estimate of time trends in some areas, we compared data from *Cancer Incidence in Five Continents* with the few available studies that measured incidence using comparable methods. With respect to risk factors, a systematic PubMed search identified 1585 relevant articles published between 1980 and 2021. Abstracts and full texts were screened. Sixty-nine eligible original cohort and case-control studies were selected. Information was extracted using a PRISMA predesigned form. Nineteen risk factors, or risk factor categories, were investigated by two or more original studies. Solitary, unreplicated studies addressed the putative role of eight more factors. Recent advances have provided further evidence supporting the carcinogenic model centred on human papillomavirus infection with different defects of the immune function. Conversely, the model centred on the role of vulvar lichen sclerosus and the often associated differentiated vulvar intraepithelial neoplasia has continued to be epidemiologically understudied. More research on the association between these two conditions and vulvar cancer is a priority.

## 1. Introduction

In the greater part of Western countries, the prognosis of patients with vulvar cancer (VC) has remained unchanged for the last two to four decades or has increased to a clinically negligible extent [1,2,3]. Even though uncommon, data showing a survival decrease over time have also been published [4]. This disappointing situation results from multiple factors common to “orphan” diseases, including—among others—the difficulty in recruiting patients for treatment trials, the lack of interest on the part of the industry to develop new effective therapies for small markets, the unavailability of specific screening techniques, the inability of healthcare systems to promote the clinical detection of VC at an earlier stage, and the absence of effective networking between primary/secondary health facilities and specialised tertiary centres [5].

Under this unfavourable clinical scenario, the key to the control of VC, which is of the squamous type in about 90% patients, is primary prevention, which involves, firstly, a better understanding of the multiplicity of risk factors associated with the development of the disease and, then, eliminating or minimizing avoidable exposures. In the presence of a permanent risk factor, when the onset of vulvar disease is not preventable, undertaking regular clinical surveillance may modify its natural history and limit the life-threatening consequences of progression. In the decision making about which preventive strategy to pursue, a consideration of geographical gradients and time trends in incidence is of help.

In fact, the epidemiologic knowledge that is needed to establish preventive measures is still incomplete. Regarding incidence, comprehensive comparisons of VC rates across countries and time have been hampered by the lack of suitable information. Most of the available data on VC are grouped within the broad category of “other female genital tumours” and, consequently, comparisons have been biased by the divergent trends of different types of cancers. Only recently, the 11th volume of *Cancer Incidence in Five Continents* [6] has presented data according to the specific *International Classification of Diseases for Oncology*, *Third Edition* (ICD-O-3) code for VC [7].

As far as the analytical epidemiology is concerned, there is a general consensus that VC arises through two distinct pathways, one associated with human papillomavirus (HPV) infection, and a second independent of it [8]. In low-income countries, the HPV-dependent disease accounts for most VC cases and affects primarily premenopausal women [9]. In high-income countries, conversely, most VCs are HPV-independent and arise in older women. The precursor of the HPV-associated VC is variously referred to as high-grade squamous intraepithelial lesion, vulvar intraepithelial neoplasia (VIN) 2/3 or usual-type VIN, whereas differentiated VIN (dVIN) is commonly considered the main precursor lesion of the HPV-independent VC. Several risk factors have been involved in the pathogenesis of both entities but with very different levels of evidence. Overall, the epidemiology of VC is still insufficiently understood, and there are multiple causes for this. The excess risk of a rare cancer is inherently difficult to demonstrate. In addition, all rare diseases are also of low interest to the general medical audience and, thus, to medical journals as well as researchers. On the other hand, however, the least studied malignancies hold a greater potential for scientific advances, with new important discoveries taking place within a short space of time. A constant and comprehensive update of knowledge offers opportunities to the practicing physicians and enables researchers to avoid redundant studies on established risk factors—unless there remain areas of uncertainty—and to direct research efforts toward the most promising hypotheses.

The above considerations formed the rationale for the present study. Our objective was to provide an updated and complete overview of descriptive and analytical epidemiology of VC. Specifically, we aimed at: (1) summarizing worldwide VC incidence rates and trends using comparable indicators; and (2) performing a systematic literature review of all known and putative risk factors for the disease.

## 2. Materials and Methods

### 2.1. Incidence

#### 2.1.1. Data Sources

The comparison of incidence data across time and populations implies that all indices are calculated with the same methods. In particular, the rates should be age-standardised in order to adjust them for the differences in age distribution across populations. To ensure meaningful comparisons, however, the age standardisation should be done with the same standard. Unfortunately, this requirement—coupled with the frequent grouping of different types of cancer—reduces the number of comparable studies.

In April 2121, we performed a literature search for incidence studies published since year 2000 (search terms: ((((vulvar OR vulva) AND (cancer OR neoplasm OR carcinoma) AND (incidence))) AND English [Language]) AND (“2000” [Date—Publication]: “2021” [Date—Publication]), and we identified only 30 articles. These studies covered 13 countries, but only three of them presented worldwide comparisons using population-based incidence data calculated with comparable methods [10,11,12]. The other studies offered occurrence data for single countries, but without comparable indicators.

In addition to the literature, the principal source of comparable incidence data is *Cancer Incidence in Five Continents*, a publication of the International Association of Cancer Registries edited by the International Agency for Research on Cancer—the specialized cancer agency of the World Health Organization. The last available edition is the 11th volume [6] which provides data from cancer registries (years 2008–2012) according to the specific ICD-O-3 code for VC, that is, C51 [7]. This publication, however, presents indicators by cancer registry and not by country. Since countries are covered wholly or partially (by local or regional cancer registries), we recalculated appropriate indicators in order to obtain meaningful comparisons.

#### 2.1.2. Methods

To investigate the time trends in VC incidence, we started with the most recent and comprehensive study, authored by Kang et al. [12], which presented age-standardised incidence rates (ASRs) in different countries and through different time periods using the World (Segi) Standard Population—as in *Cancer Incidence in Five Continents*. Kang et al. had access to the *Cancer Incidence in Five Continents* data bank, with individual records, which allowed for selecting the ICD-O-3 topography code for VC (i.e., C51) and calculating incidence rates specific for the disease. Furthermore, they regrouped cancer registries by country and continent, showing results according to more meaningful geographic aggregates. We also used the study of Bray et al. [10] to add more countries for comparison. Since Bray et al. did not provide indicators grouped according to the same periods as in the study by Kang et al., we derived ASRs for the median year of these periods using Figure 3 from the article of Bray et al. Finally, we calculated age-standardised incidence data for the last period available in *Cancer Incidence in Five Continents*, vol. XI, i.e., 2008–2012, for the same areas taken into consideration in the studies of Kang et al. and Bray et al. This was facilitated by the online analysis tool made available at the website of the International Association of Cancer Registries www.iacr.fr (last accessed 5 January 2022).

### 2.2. Risk Factors

#### 2.2.1. Literature Search Strategy

A systematic search of PubMed was performed in April 2021 in order to identify all relevant articles published in English since January 1980. The following search terms were used: ((((vulvar OR vulva) AND (cancer OR neoplasm OR carcinoma) AND (risk))) AND English [Language]) AND (“1980” [Date—Publication]: “2021” [Date—Publication]).

The search was repeated using a different string, characterised by the inclusion of the MESH term ‘Neoplasms’ in order to improve the sensitivity of the procedure, but with equal results: ((((vulvar OR vulva) AND (cancer OR neoplasm OR carcinoma OR Neoplasms [MeSH Terms]) AND (risk))) AND English [Language]) AND (“1980” [Date—Publication]: “2021” [Date—Publication]).

#### 2.2.2. Study Selection

The abstracts of all studies retrieved were independently screened by a pair of reviewers (L.B. and S.F.) according to a predetermined list of inclusion and exclusion criteria. The inclusion criteria were as follows: (1) article reporting an original study or a systematic review or a meta-analysis addressing the association between epidemiologic risk factors and primary VC (topography code C51 according to the IC-D-O, third ed. [7]; (2) article providing a quantitative estimate of the association as obtained using a cohort or a case-control control approach; and (3) article in English.

**Figure 1:**
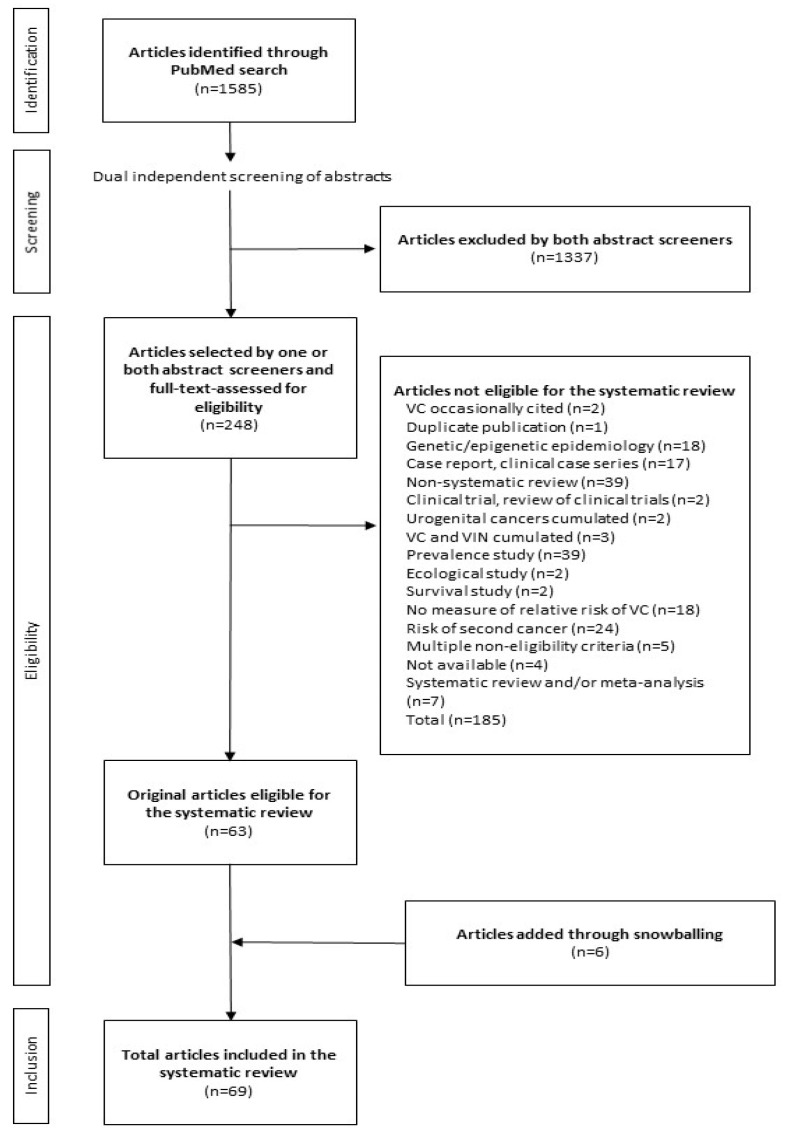
PRISMA flow diagram of identification, screening and inclusion of articles. VC indicates vulvar cancer. VIN indicates vulvar intraepithelial neoplasia. Systematic literature reviews and meta-analyses, albeit not formally evaluated, were selected with standard methods to be briefly presented in the article. ‘Not available’ indicates an article potentially eligible but not retrieved in full text. ‘Risk of second cancer’ indicates risk of VC for patients previously diagnosed with another type of cancer.

The exclusion criteria can be drawn from the largest box in Figure 1, which depicts the flow diagram of the PubMed search. The box shows the distribution of articles not eligible for the systematic review according to the cause for noneligibility. The articles reporting systematic literature reviews and meta-analyses, albeit not formally evaluated nor taken into account to draw up the conclusions of the review, were selected with standard methods to be briefly presented here for reasons of completeness of information.

Disagreements as to article eligibility were resolved by discussion and final consensus. If a consensus was not reached, the full text of the article was independently reviewed by both screeners to determine whether it fitted the inclusion criteria. Again, differences of opinion were resolved through discussion and final consensus between them. The reasons for exclusion were recorded. In addition, the same reviewers evaluated the abstracts of the references listed in all included articles in order to identify additional titles (a technique called snowballing). The same methods as above were used. When multiple reports from a single study were selected in this way, the most recent results based on the largest number of patients were included, unless different outcomes were reported. This sub-selection was based on discussion between the two reviewers.

Some studies ineligible for the review but containing scientific arguments and supporting data of interest are cited in the discussion section of the manuscript.

#### 2.2.3. Data Extraction

In order to produce a summary of eligible studies, a systematic approach to data extraction was used. After a pilot test, the following information was extracted by one of us (L.B.) with a standard form: risk factor investigated, first author’s last name, publication year, country, study design, study population, number of cases and controls (for case-control studies), number of women exposed and incident cases (for cohort studies), age at entry, type of comparison, target disease (VC not otherwise specified, VC; vulvar squamous cell carcinoma, VSCC; vulvar/vaginal cancer, V/VC; vulvar/vaginal squamous cell carcinoma, V/VSCC), quantitative estimate of the association, and adjusting variables used. The integrity of data was subsequently checked by S.F. and disagreements were resolved by discussion between the two reviewers. No authors of original articles were contacted for additional study information. The original statistical terminology was not modified.

#### 2.2.4. Data Analysis

The characteristics and results of the articles selected were narratively summarised by risk factor and risk factor category. Risk factors were categorised in an arbitrary manner. In the results section, the 95% confidence intervals (CIs) that appear in the tables are not repeated in the text. If possible, the age of subjects studied was expressed in completed years.

The results of previous literature reviews and meta-analyses were separately evaluated and briefly reported. The review was conducted in accordance with the preferred reporting items for systematic review and meta-analyses (PRISMA) statement [13].

## 3. Results

### 3.1. Incidence

Sankaranarayanan et al. [11] reported summary statistics indicating some international differences in the incidence of VC. Bray et al. [10] reported a more in-depth assessment of international variation in the incidence rates of VC as well as vaginal cancer in 2008–2012. The data were contributed by cancer registries in 68 countries. The study also assessed the time trends in incidence in eight countries (Australia, China, Colombia, India, Norway, Slovakia, the US, and the UK) over the period 1983 to 2012. There was a 30-fold variation in incidence rates, with the highest ones being found in the data from South Africa (ASR, 7.2 per 10,000). High rates were also seen in specific countries of Europe and North America. The second highest incidence worldwide was in Germany (ASR, 4.2 per 10,000). Conversely, the disease was rare in western Asia and the Middle East (ASR, <0.2 per 10,000 in Bahrain, Kuwait, Saudi Arabia and Qatar). An increasing incidence trend was found in the data from Australia, Norway, UK, and Slovakia. The rise was more rapid for women aged < 60 years at diagnosis. For these, the estimated annual percentage change over the last decade covered by the study varied between 1.7% in Norway to 4.1% in Slovakia. The incidence increase tended to be greater at ages < 60 in the majority of the eight countries with trend data. At variance with this pattern, the magnitude of the incidence increase in the US was similar between the two age categories.

Kang et al. considered 13 high-income countries with cancer registry data available for the whole time period 1988–2007, i.e., Canada, US, nine European countries, Australia and Japan [12]. During the study period, the 5-year average percent incidence change was 4.6% in women of all ages, and 11.6% in those aged < 60 years. No change was observed in women aged 60 years or older. The standardised incidence rate ratio for 2003–2007 vs. 1988–1992 was 1.38 (95% CI, 1.30–1.46) but not in older women (standardised incidence rate ratio, 1.01; 95% CI, 0.97–1.05). The increase in incidence in women < 60 years of age caused a significant increase in overall incidence (standardised incidence rate ratio, 1.14, 95% CI, 1.11–1.18).

Incidence time trends were then explored contrasting the data obtained as described above. Table 1 summarises the comparison. In general, VC incidence increased more rapidly in the last recorded period (2008–2012), while being substantially stable previously. The incidence increase was observed worldwide, in western countries as well as in Asia, with the exception of Colombia (Cali) and India (Chennai). The most striking increase was observed in Saarland (Germany), where the rate rose by four times from the first period (1988–2002), when incidence was substantially stable, to the last one (2008–2012), when the ASR grew up to 5.7 cases per 100,000 inhabitants.

### 3.2. Risk Factors

Figure 1 depicts the PRISMA flow diagram of the PubMed search. The number of articles identified was 1585. After dual independent screening of all abstracts, 1337 studies were excluded. The remaining 218 studies were selected by at least one screener and underwent full-text assessment. This led to the exclusion of 185 studies. This number included four original articles which were considered potentially eligible based on the abstract but could not be retrieved in full text (see Section 4.4), and seven articles reporting systematic literature reviews and meta-analyses [14,15,16,17,18,19,20], briefly presented here (see Section 4.6). The number of original articles selected was 63. Six more original articles were identified through snowballing for a total of 69 articles [21,22,23,24,25,26,27,28,29,30,31,32,33,34,35,36,37,38,39,40,41,42,43,44,45,46,47,48,49,50,51,52,53,54,55,56,57,58,59,60,61,62,63,64,65,66,67,68,69,70,71,72,73,74,75,76,77,78,79,80,81,82,83,84,85,86,87,88,89]. In the references section, in order to help the reader to locate rapidly each referenced study, these 69 publications are sorted alphabetically.

The following 19 risk factors, or risk factor categories, were investigated by two or more original studies: HPV infection; familial clustering of HPV-associated cancers; other sexually transmitted diseases; sexual behaviour; cervical intraepithelial neoplasia grade 1–3 (CIN1-3); vulvar lichen sclerosus (VLS); autoimmune diseases, including systemic lupus erythematosus (SLE) and rheumatoid arthritis; menstrual and reproductive factors; oral contraceptive and menopausal hormone use; metabolic syndrome, diabetes, and body mass index (BMI); food items; alcohol consumption; smoking; human immunodeficiency virus and acquired immunodeficiency syndrome (HIV-AIDS); solid-organ transplantation; breast implants; Fanconi anaemia; previous abnormal cervical cytology; and education.

Solitary, unreplicated studies addressed the putative role of eight more risk factors, for a total of 27. These uncommon risk factors included deprivation index; seafaring work; vulvar lichen planus; husband’s cancer of the penis; psoriasis; allergies; leucoplakia and inflammation; and inflammatory bowel disease.

The number of original articles published was one in 1980–1989, 16 in 1990–1999, 19 in 2000–2009 and 33 in 2010–2020 (no publications in January–April 2021), for an average annual number of 0.1, 1.6, 1.9, and 3.3. The median year of publication was 2008. Thirty-six (52%) articles were from the European countries, 28 (41%) from northern and southern America, and five (7%) from Asia, Oceania, and Africa. Cohort studies (*n* = 54) accounted for an average 78% articles, but the proportion increased from 7/17 (41%) in the years 1980–1999 to 47/52 (90%) in the last two decades.

Table 2, Table 3, Table 4, Table 5 and Table 6, all subdivided in two panels, show a summary of eligible studies grouped according to the risk factor or risk factor category investigated. More precisely, studies on HPV infection, familial clustering of HPV-associated cancers, other sexually transmitted diseases and sexual behaviour are shown in Table 2. Table 3 shows the studies on CIN1-3, VLS, SLE, rheumatoid arthritis and part of menstrual and reproductive factors. Table 4 considers the remaining menstrual and reproductive factors as well as the studies addressing oral contraceptive and menopausal hormone use, metabolic syndrome, diabetes, BMI, food items and alcohol consumption. Studies dedicated to investigating the role of smoking, HIV-AIDS and solid-organ transplantation are shown in Table 5. Finally, Table 6 lists the studies concerning breast implants, Fanconi anaemia, previous abnormal cervical cytology, and education. For each risk factor, the articles are sorted by year of publication.

Solitary studies dealing with uncommon risk factors, not included in Table 2, Table 3, Table 4, Table 5 and Table 6, are briefly dealt with below (see Section 3.2.20).

#### 3.2.1. HPV Infection

In 1997, Bjørge et al. designed a case-control study on the role of HPV infection in noncervical anogenital cancers [24]. The study was nested within two serum bank cohorts, the Finnish population-based maternity cohort (including virtually all pregnant women in Finland, 1983–1993) and the Janus Project cohort (people undergoing preventive blood tests and blood donors from several Norwegian counties, 1973–1997). At a cut-off point of 0.100 absorbance units, the odds ratio (OR) of V/VC was 5.5 for women infected with HPV type 16 and 1.5 (95% CI, 0.3–7.5) for those infected with HPV type 18. At a cut-off point of 0.239, the OR for women with HPV type 16 was 4.5 (95% CI, 1.1–22.0).

A seroepidemiologic case-control study, with limited statistical power, associated HPV-16 seropositivity with an OR for VC of 2.9 at a borderline level of significance [52]. Subjects with high antibody levels had a 20-fold increased risk of disease (OR, 20.1; 95% CI, 5.4–76.7). The association with HPV-16 seropositivity was stronger for women diagnosed with the warty/basaloid type of VC (OR, 3.8; 95% CI, 0.76–18.9) than for those diagnosed with the keratinizing VSCC (OR, 1.6; 95% CI, 0.35–7.4). The risk associated with HPV-16 seropositivity was higher among smokers (OR, 8.5; 95% CI, 3.8–19.0) than among nonsmokers (OR, 3.4; 95% CI, 0.85–13.0).

In the case-control study authored by Madeleine et al., HPV-16 seropositivity conveyed an OR for VSCC of 2.8, with no significant effects being observed among HPV-18, HPV-6 and HPV-2 seropositive women [59].

Another case-control study was nested in the European Prospective Investigation into Cancer and Nutrition cohort study [57]. With a prevalence of 26.9% among VC cases vs. 9.9% among controls, the seropositivity against the HPV16 LI protein conveyed a significant increase in the risk of disease (OR, 3.4). The seropositivity against other HPV proteins and genotypes had no demonstrable effects.

#### 3.2.2. Familial Clustering of HPV-Associated Cancers

In a study of familial clustering of HPV-associated cancers, a cohort of 3,625,784 female offspring in Sweden, identified between 1958 and 2004, was followed-up until 2004. The risk of VSCC for female offspring was found to be greater when a sister (standardised incidence ratio (SIR), 1.80) or the mother (SIR, 1.76) were affected by cervical squamous cell carcinoma [53]. The study was subsequently updated (1958–2015) using the risk of cumulated V/VC as an endpoint. When a family member was affected by anal cancer and V/VC, the SIR was 2.38 and 2.72, respectively [89].

#### 3.2.3. Other Sexually Transmitted Diseases

The studies on the role of a history of genital warts, a proxy of exposure to high-risk HPV, have yielded consistent results. Brinton et al. reported an OR for VC of 14.55 [29]. A case-control study by Sherman et al. provided a closely comparable estimate for VSCC, i.e., 17.3 [78]. This is also the case for a cohort study of Danish women hospitalised for genital warts, in which Blomberg et al. observed an overall SIR for VC of 14.8 [26]. The incidence ratio, in fact, decreased with increasing length of follow-up, that is, from 90.6 (95% CI, 49.5–152.0) in the first year to 11.0 (95% CI, 7.7–15.3) ≥ 10 years after diagnosis. In the case-control study authored by Madsen et al., a history of anogenital warts conveyed an almost sixfold increased risk of VSCC [61].

Friis et al. reported on a nationwide Danish cohort of women hospitalized for condylomata acuminate [45]. The SIR for VC was 40.1, with a marginal difference between women aged <40 years and ≥40 years. In a Swedish cohort of comparable size, Nordenvall et al. observed a SIR for VC of 10.2 [65].

#### 3.2.4. Sexual Behaviour

The indicators of sexual behaviour have not been consistently associated with the risk of VC. Mabuchi et al. conducted a case-control study on patients from 115 hospitals in five US metropolitan areas [58]. Coital experience and age at first coitus were not significant determinants of the risk of VC. Regarding the age at first coitus, Sherman et al. obtained similar results for VSCC [78]. Conversely, they found a strong association between the total number of sexual partners, the indicator of sexual behaviour most often used in the relevant literature, and the risk of VSCC (OR, 8.2 for ≥15 partners vs. 0–1). This risk factor was confirmed by Hildesheim et al. using the risk of VC as an endpoint (OR, 3.4 for ≥3 partners vs. 0–1) [52] but not by Brinton et al. [29], Parazzini et al. [68], and Madsen et al. [61]—the latter focusing on VSCC. The number of marriages, too, was the object of conflicting results [58,78]. In the study by Mabuchi et al., the age at first marriage was a moderate risk factor (OR, 3.29 at age ≥30 vs. <20) [58].

Madsen et al. investigated other indicators of sexual behaviour [61]. The practice of anal intercourse and that of genital washing before and after sex did not exert significant effects on the risk of VSCC. The study also addressed some partner-related sexual factors. Lifetime number of other sexual partners and history of anogenital warts were not significantly associated with the risk of VSCC. Conversely, having a male sexual partner who is unmarried and without current male partners was shown to be a strong protective factor (OR, 0.20).

#### 3.2.5. CIN1-3

Most studies on the role of a history of cervical intraepithelial disease have considered cohorts of women with CIN3. A cohort of Norwegian women experienced a SIR for V/VC of 4.04 [25]. A virtually equal result was obtained for VC in a study conducted in south-east England [42]. In a Swedish cohort, with a median follow-up of 27 years, the incidence rate ratio (IRR) for VC was 2.22 [40]. A greater incidence was observed during the first year after recruitment (IRR, 5.97; 95% CI, 1.85–13.94). The excess risk was inversely related to age. For women aged 18–29 years at entry, the IRR was 23.32 (95% CI, 5.38–101.01). For women aged ≥60 years, the risk increase was modest but still significant (IRR, 1.52; 95% CI, 1.07–2.17).

In the Million Women study, involving 1.3 million women who participated in the UK national breast screening programme between 1996 and 2001, the registration of CIN3 before recruitment was associated with a relative risk (RR) of VC of 2.68 [33]. A nearly equal result was obtained by Pan et al., who studied the risk of cervical and noncervical HPV-associated cancers in a cohort of Scottish patients [67]. The SIR for VC was 2.8. The highest overall increase in the risk of VC was found in a Dutch cohort, with an IRR of 4.97 [39]. Patients were followed-up for 25 years. In the first year after recruitment, the IRR reached the level of 14.94 (95% CI, 1.98–112.98).

Studies encompassing lower grades of CIN, that is, CIN1-3 [55,56] or CIN2-3 [40,71], confirmed the above observations with a single exception, an Italian hospital-based cohort study of patients with CIN2-3 [71] in which the incidence increase was moderate and not significant. A study reported from the British Columbia associated a diagnosis of CIN2-3 with a SIR for VC of 2.90 [47]. The excess risk was concentrated in women with a history of CIN3, with a SIR of 3.79 (95% CI, 2.41–5.69). A history of CIN2 did not convey a significant effect (SIR, 1.47; 95% CI, 0.67–2.79). The SIR was inversely related to the length of follow-up (0.5–4 years, 12.1; 95% CI, 9.5–15.2).

#### 3.2.6. VLS

In a Finnish nationwide cohort study by Halonen et al., a history of VLS was associated with a SIR for VSCC of 33.6 [48]. The risk was greater during the first year of follow-up (SIR, 140; 95% CI, 108–177) and among women diagnosed with VLS during their 30 s (SIR, 385; 95% CI, 122–928).

A high SIR for VC, 39.58, was also reported from Italy by Corazza et al. [34]. The excess risk was slightly greater for women aged ≥ 70 years (SIR, 46.62; 95% CI, 15.14–108.80). In this small study, the attributable risk of VC cancer due to VLS was estimated to be 98%.

#### 3.2.7. Autoimmune Diseases

In a cohort of women with SLE, Mellemkjaer et al. found a RR of V/VC of 5.7 [62]. Parikh-Patel et al. followed-up a cohort of Californian patients and estimated a SIR for V/VC of 3.27 [69]. For the same disease, a study from Taiwan reported a SIR of intermediate level, 4.76 [31].

Dreyer et al. investigated a multihospital-based cohort and observed a high SIR for cumulated V/VC, 9.1 [38]. In a multicentre international cohort study by Bernatsky et al., focusing VC, an estimate of 3.78 was obtained [22].

Two studies evaluated cohorts of patients with rheumatoid arthritis. Parikh-Patel et al. considered a state-wide population-based cohort of women hospitalised for the disease, and reported no change in the risk of V/VC (SIR, 0.99) [70]. In a population-based cohort of Taiwanese women registered in a health insurance database, a moderate excess risk of V/VC cancer was observed (SIR, 1.69) [32].

#### 3.2.8. Menstrual and Reproductive Factors

Several studies addressed the association between menstrual and reproductive factors and the risk of VC or, in certain analyses, VSCC. Virtually no evidence was found. Negative results were obtained for age at menarche [30,33,58], pregnancy [79], age at first pregnancy [58,79], number of pregnancies [58,79], parity [33,68,79], age at first (live) birth [30,79], number of (live) births [30,79], and menopausal status [58,68].

A younger age at menopause was associated with the risk of VSCC in one study [33] but not in others [30,58]. Sherman et al. associated a history of induced abortion with an increased risk of VSCC of borderline statistical significance [79]. According to three studies [30,33,79], women reporting a history of miscarriage, tubal ligation, and hysterectomy have no significant variations in the risk of VC and VSCC.

#### 3.2.9. Oral Contraceptive and Menopausal Hormone Use

Brinton et al., Coffey et al., and Sherman et al. found no significant association of oral contraceptive use with the risk of VC [30,33] and VSCC [79]. Brinton et al. and Coffey et al. reported equally negative results for menopausal hormone use [30,33]. The Million Women study [33], however, showed a RR of 1.52 (95% CI, 1.22–1.89) for women aged <50 years at menopause or oophorectomy who never used hormone therapy.

#### 3.2.10. Metabolic Syndrome, Diabetes, BMI

Nagel et al. reported on the association between metabolic syndrome and the risk of VC [64]. The study was based on regional cohorts from three European Countries. The metabolic syndrome conveyed an increased risk of VC, with a hazard ratio (HR) of 1.78 for one standard deviation increment in the standardised z-scores. With respect to the components of metabolic syndrome, the HR was 1.98 for blood glucose concentration and 2.09 for triglyceride concentration. There was no evidence for an association of serum cholesterol levels as well as blood pressure with the risk of VC.

Coffey et al. (Million Women Study) and Brinton et al. found no significant association between diabetes and the risk of VC [30,33].

A risk increase for women in the highest BMI category (OR, 2.9) as compared with the lowest one was found in a case control study by Sherman et al., which considered VSCC alone [79]. Parazzini et al. found a significant OR of 2.5 for VC when comparing women with a BMI of 25.4–28.1 as well as those with a BMI ≥ 28.2 with women in the reference category < 21.3 [68]. Nagel et al. observed a 1.36 HR for VSCC for one standard deviation increment in standardised z-score of BMI [64]. In the Million Women study, both a BMI of 25.0–29.9 (RR, 1.19) and >30.0 (RR, 1.71) predicted an increased risk of VC vs. a BMI < 25.0 [33]. Obese women (BMI > 30.0) had a particularly elevated risk of VSCC (RR, 1.84; 95% CI, 1.53–2.21), with little or no increased risk for basal cell, glandular, and melanocytic tumours. Brinton et al. reported a 62% increase in the risk of VC for women with a BMI ≥ 30.0 vs. < 25.0 [30]. Among patients with VSCC alone, the association was stronger (OR, 2.15; 95% CI, 1.30–3.57) and women with a BMI of 25.0–29.9, too, had a risk increase (OR, 1.58; 95% CI, 0.97–2.55).

#### 3.2.11. Food Items

Aside from alcohol consumption, food items were seldom investigated. In a study by Mabuchi et al., 3–4 and ≥5 cups of coffee per day were associated with an OR of 2.99 and 2.42 for VC [58]. In Italy, Parazzini et al. failed to confirm this finding [68] but reported an OR of 2.0 for women eating < 7 portions of green vegetables per week vs. women eating ≥ 14 portions. The trend associated with carrot consumption was similar but not significant.

#### 3.2.12. Alcohol Consumption

Parazzini et al., Weiderpass et al., Brinton et al., and Coffey et al. observed no significant association between alcohol consumption and the risk of VC [30,33,68] and VSCC [87]. In the case-control study of Madsen et al., conversely, women reporting no alcohol consumption had an over 60% drop in the risk of VSCC [61].

#### 3.2.13. Smoking

Mabuchi et al. related a current number of 10–20 cigarettes smoked per day to an OR of 2.46 for VC [58]. Brinton et al. observed a nonsignificantly elevated risk [29]. In 2017, however, a second study from the same first author reported a 86% risk increase for current vs. never smokers [30], with a stronger association among patients with VSCC (OR, 2.55; 95% CI, 1.53–4.27) and no effect among patients with non-VSCC.

In the study by Daling et al. [36], current and former smokers had an OR for VSCC of 4.8 and 1.8, respectively. There was no clear trend to increasing risk with increasing number of years smoked (not shown in Table 5). Conversely, the risk increased with increasing number of cigarettes/day and was inversely related to the age at start of smoking. Interestingly, the presence of both genital warts and smoking was associated with a risk of VSCC largely greater than one would expect if the effects of the two exposures were additive. Compared with nonsmokers free of genital warts, nonsmokers with genital warts had an OR of 7.8 (95% CI, 3.6–17.3). Smoking alone was associated with an OR of 4.2 (95% CI, 2.8–6.4). When both exposures were present, the OR rose to 51.3 (95% CI, 26.1–100.8).

In the study by Madeleine et al., ever and current smokers had an OR for VSCC of 2.2 and 3.0, respectively, vs. never smokers [59]. The ORs were higher among HPV16 seropositive women. In particular, seropositive current smokers had an OR of 18.8 (95% CI, 11.9–29.8) vs. an OR of 4.9 (95% CI, 3.3–7.5) for seronegative current smokers. Among seropositive women, a number of cigarettes/day smoked ≥ 20 (vs. <10) was associated with an OR of 25.1 (95% CI, 14.7–42.6), whereas seronegative women had an OR of 5.6 (95% CI, 3.6–8.9).

Among current smokers, Madsen et al. observed an OR for VSCC of 2.61 vs. lifelong non-smokers [61]. Interestingly, the role of smoking was restricted to high-risk HPV-positive VC cases (OR, 2.79; 95% CI, 1.30–5.99). No effect was found among high-risk-HPV-negative VC cases (OR, 1.03; 95% CI, 0.36–2.94).

In addition to the study by Brinton et al. mentioned earlier [29], negative results were also obtained by Parazzini et al. [68] and Coffey et al. [33], the latter focusing on VSCC. These two research groups observed virtually no increase in the risk of disease for ever and current smokers vs. never smokers.

#### 3.2.14. HIV-AIDS

In a study involving HIV-infected members of the Kaiser Permanente health care delivery system, Silverberg et al. observed an almost 20-fold increased risk of V/VC [80]. A more pronounced risk increase, with a SIR for VC of 69.2, was observed in a hospital-based cohort of Italian HIV-infected women [44]. In a multistate cohort study from the US, the risk increase, 9.35, was more moderate [50], although the SIR for VC was threefold greater for women with AIDS (12.30; 95% CI, 10.26–14.62) than for women with HIV only (4.00; 95% CI, 2.54–6.00). Mpunga et al. reported a hospital-based case-control study from Rwanda. For HIV-infected women, the OR for VC was 17.8 [63].

An increased risk of VC among HIV-infected Hispanic women was reported by a large cohort study from the US [66], which compared Hispanic patients with the general Hispanic population. The SIR was 9.03. However, when directly compared with HIV-infected non-Hispanic whites and non-Hispanic blacks, HIV-infected Hispanic women showed a lower risk of VC. The IRR was 0.40 (95% CI, 0.24–0.67) and 0.62 (95% CI, 0.41–0.95), respectively. It must be noted that, at variance with this pattern, HIV-infected Hispanic women retained a higher risk of cervical cancer than non-Hispanic whites (IRR, 1.70; 95% CI, 1.19–2.43).

Hessol et al., in a study from the San Francisco area, included subjects registered with HIV as well as AIDS [51]. They reported a SIR for VC of 13.3. In a cohort study of people with AIDS in the US, Frisch et al. observed a RR of V/VSCC of 5.8 [46]. The excess risk of disease was much higher for women aged < 30 years at the onset of AIDS (RR, 37.2; 95% CI, 7.7–108.8). Also, the RR was higher for Hispanic women (15.5; 95% CI, 5.7–33.7).

Tanaka et al. studied the incidence of cancer in a population-based cohort of people diagnosed with AIDS in São Paulo (Brazil) [83]. The reporting article did not provide the absolute number of women, who accounted for 30% of the total 480,102 person-years at risk. The SIR for V/VC was 6.78.

#### 3.2.15. Solid-Organ Transplantation

In the earliest study on the risk of VC among renal transplant recipients, Fairley et al. found an over 50-fold increase [43]. In a parallel cohort of patients undergoing chronic dialysis, the excess incidence of VC was not significant. Skov Dalgaard et al. studied a Danish cohort of women treated with renal replacement therapy (i.e., transplantation or chronic dialysis) [82]. The IRR for V/VC was 5.81. Transplant recipients had a 3.31-fold (95% CI, 1.13–9.69) greater risk than patients treated with dialysis. Three other cohort studies confirmed the existence of a huge increase in the risk of VC [84], VSCC [73] and V/VC [23] for women with renal transplant. The estimate by Villeneuve et al. (SIR for VC, 5.5) was considerably lower [86].

A marked variability of results was also observed in studies pooling patients with renal transplant and other solid-organ transplants. In a Swedish cohort (renal transplant recipients, 84%) by Adami et al., the SIR for VC was 26.2 [21]. For the same disease, Engels et al. (renal transplant recipients, 58%) reported a manifold smaller increase (SIR, 7.60) [41]. This was fully confirmed by a larger US cohort study of solid-organ transplant recipients (renal transplant recipients, 59%) reporting an IRR for VC of 7.3 [60]. In this study, time since transplantation was directly associated with the risk of VC. After five years, the IRR was 2.1 (95% CI, 1.5–2.9) (reference category, <2 years). Only patients treated with azathioprine were demonstrated to be at increased risk of VC (IRR, 2.0; 95% CI, 1.2–3.2).

In a small Swedish study, a cohort of paediatric solid-organ transplantation patients (renal transplant recipients, 62%) was studied. The SIR for V/VC was as high as 665.0 [81]. In a study addressing the same type of patients (renal transplant recipients, 44%), the increase in the risk of VC was much less pronounced (HR, 17.4) but still significant (the 95% CI was provided only graphically) [88].

A cohort study of liver transplant recipients was reported by Schrem et al. They observed a SIR for VC of 23.80 [77].

#### 3.2.16. Breast Implants

Two multicentre hospital-based cohort studies explored the risk of cancer in women with cosmetic breast implants. Brinton et al. found a SIR for V/VC of 2.51 [28]. Deapen et al., conversely, found a nonsignificantly increased risk of VC [37].

#### 3.2.17. Fanconi Anaemia

Two small cohort studies considered clinical series of patients with Fanconi anaemia [74,75]. There were 69 and 78 female patients, respectively. Three of these, in both studies, were diagnosed with VC, with a SIR of 4317 and 2411 (indicated as significant in the article), respectively.

#### 3.2.18. Previous Abnormal Cervical Cytology

Brinton et al. found that a previous abnormal Pap smear did not increase the risk of VC to a significant extent (OR, 1.41) [29]. Interestingly, they observed a greater increase for women with no previous history of cervical disease (OR, 2.46; 95% CI, 0.9–6.7). For women with previous abnormal Pap smear, Sherman et al. found an OR for VSCC of 5.0 [78]. Viikki et al. studied a cohort of Finnish women with abnormal cervical cytology result (Papanicolaou classes III–IV) and subsequent negative histologic assessment [85]. The risk of VC was significantly increased (SIR, 5.8). The risk peaked during the second to fourth year of follow-up (SIR, 11.3; 95% CI, 1.4–41.0).

#### 3.2.19. Education

Parazzini et al. demonstrated a nonsignificant 50% decrease in the risk of VC for women with ≥12 years of school [68]. A similar but significant effect was reported by Madsen et al. in association with ≥10 years of school [61]. The estimate was restricted to VSCC.

#### 3.2.20. Uncommon Risk Factors

Some other potential risk factors have been the subject of single anecdotal observations (not presented in Table 2, Table 3, Table 4, Table 5 and Table 6). In the Million Women study, the deprivation index did not predict the risk of VC [33].

A cohort study of 11,529 Finnish seafarers (98% <60 years old) showed an increase in the risk of V/VC (*n* = 7) after 20 years since first employment (SIR, 4.2; 95% CI, 1.7–8.7) [72]. The estimate was adjusted for age.

In a Finnish nationwide, population-based cohort of 13,100 women (81% ≥50 years old) diagnosed with lichen planus, Halonen et al. observed a doubling of the incidence of VC (*n* = 18) (SIR, 1.99; 95% CI, 1.18–3.13) [49]. The estimate was adjusted for age, calendar period, and follow-up period. The risk was higher in the first year of follow-up (SIR, 8.27; 95% CI, 2.69–19.3).

Iversen et al. compared a cohort of wives of 423 men with squamous cell carcinoma of the penis with a control cohort of wives of 444 unaffected men [54]. They observed one and zero cases of VSCC, respectively.

In a Swedish population-based cohort of 4467 women (mean age, 50) hospitalised for psoriasis, the SIR for VC (*n* = 6) was 3.24 (95% CI, 1.18–7.06) [27]. The estimate was adjusted for age and calendar year.

In a case-control study on the association between allergies and the risk of cancer, the presence of allergic rhinitis was associated with a decreased risk of V/VC (OR, 0.79; 95% CI, 0.71–0.87) [35]. The estimate was adjusted for age, race, socioeconomic status, number of visits per year, and history of chronic obstructive pulmonary disease. Asthma and eczema had no effects on the risk of V/VC.

In a study mentioned above, Mabuchi et al. found an association between a history of leucoplakia and inflammation (not otherwise specified) of the vulva with the risk of VC at a level of significance of *p* < 0.005, although they did not provide an estimate of the excess risk [58].

In a small population-based cohort of 55 patients (age ≥ 18) with inflammatory bowel disease, Rouvroye found a SIR for V/VC not significantly greater than the unity [76].

## 4. Discussion

### 4.1. Research Trends

Over the years, the number of formal epidemiologic studies on VC has grown. Some analytical studies of unprecedented size, recently published, have been particularly important in broadening our understanding of the aetiology of the disease and our ability to identify persons at high risk [30,33,64]. The ongoing upward incidence trend is expected to attract further attention from researchers. The increase in scientific publications, however, has only been driven by the research on the carcinogenic model centred on HPV infection and on different defects of the immune function. Conversely, the epidemiologic evidence for the model centred on the role of VLS and dVIN is still inconclusive.

### 4.2. Incidence

International incidence comparisons at the global level are hampered by the fact that a number of national and regional studies have not used the same methods for age-standardisation. For this reason, we selected only three studies on incidence gradients and trends reporting data age-standardised to the World Standard Population [10,11,12]. The two largest ones, both using data from the International Association of Cancer Registries, were authored by Bray et al. [10] and Kang et al. [12]. Overall, the study years were 1988 to 2007. Bray et al. reported a 30-fold variation in incidence rates. The highest ones were found in the data from South Africa. An increasing incidence trend was found in several countries, which was more rapid for women aged < 60 years at diagnosis. The incidence increase tended to be greater at ages < 60 in the majority of the eight countries with trend data.

Kang et al. considered 13 high-income countries with cancer registry data available for the whole time period 1988–2007 [12]. The 5-year average percent change was 4.6% in women of all ages, and 11.6% in those aged < 60 years. No change was observed in women aged 60 years or older. Both groups of researchers interpreted their findings to be consistent with changing sexual behaviours and increasing levels of exposure to the HPV infection in cohorts born around/after about 1950. When comparing these two studies with the more recent data from *Cancer Incidence in Five Continents*, vol. XI (2008–2012), it appeared that the incidence increase was more rapid in the last time period.

Other researchers have explored incidence time trends, but within single countries (or regional areas) and without indicators made comparable across studies. Beyond this problem, increasing time trends have been reported from many studies in Western countries [3,4,90,91]. Only in a few populations have the rates shown a stable or nonsignificantly increasing trend [92]. In general, the incidence increase has been confirmed to be restricted to women below the age of 50–60 years [3], which further supports the view that changes in sexual behaviour have led to increasing levels of exposure to the HPV infection in recent birth cohorts [12].

### 4.3. Comments on Selected Risk Factors and Risk Factor Categories

#### 4.3.1. HPV, Sexually Transmitted Diseases, Sexual Behaviour

Four serologic case-control studies have associated HPV 16 seropositivity with a three- to fivefold increased risk of VC [52,57], VSCC [59], and V/VC [24]. No evidence for a comparable effect by HPV 18 seropositivity was reported. Six other studies have consistently associated a history of anogenital warts [26,29,61,78] and condylomata acuminata [45,65], proxies of exposure to high-risk HPV, with a considerably larger risk increase for VC and VSCC—generally greater than 10-fold. Clinical research has shown that defects in the immune function, particularly in cell-mediated immunity, play an important role in the occurrence, development and recurrence of these conditions [78]. The number of sexual partners, another proxy of exposure to high-risk HPV, has been associated with the risk of VC by two of the four studies investigating this association [52,78]. One study failed to demonstrate a significantly increased risk for women reporting current or past sexual partner(s) with genital warts [61].

A key observation was reported by Daling et al., that is, a 50-fold increase in the risk of VSCC when genital warts and smoking were both present [36].

#### 4.3.2. Familial Clustering of HPV-Associated Cancers

A different, and original, support to the etiologic role of HPV infection in vulvar carcinogenesis was provided by a Swedish study of familial aggregation of HPV-associated cancers. The results showed an increased risk of VSCC for female offspring when a sister or the mother were affected by cervical squamous cell carcinoma [53]. In an update of the study (that we have not considered a duplicate publication because the outcome measures were different), an increased risk of V/VC was observed when a family member was affected by anal cancer and V/VC [89]. As HPV infections are usually sexually transmitted, a familial increase in the risk of V/VC would suggest sexual abuse situations. More likely (or more frequently), the findings of the above studies are the result of shared environmental and genetic factors. They may include cigarette smoking and inherited variation in genes regulating the immune response to HPV and/or tumour development [53].

#### 4.3.3. CIN1-3

A history of CIN3 is an established risk factor for VC, which further reinforces the role of high-risk HPV infection in vulvar carcinogenesis. All of the six studies reviewed indicated a consistent two- to fivefold excess risk of VC [33,39,40,42,67] and V/VC [25]. The similarity of their results with the above ones for HPV infection indicates their overall robustness.

Four more studies considered the risk of VC for women with a history of CIN2-3 [47,71] and CIN1-3 [55,56]. Preti et al. reported a moderate and nonsignificant risk increase for CIN2-3 patients [71]. A study from the British Columbia showed that the increase was concentrated, in fact, among women with a history of CIN3 [47]. At variance with these observations, two studies of women reporting a history of CIN1-3 confirmed a SIR for VC of approximately 5 [55,56].

The association of previous diagnosis of CIN3 with the risk of VC is prone to confounding factors—most often only partially adjusted for—as well as biases. Theoretically, the latter may act in opposite directions. On the one hand, current electrosurgical treatments of CIN3 are less invasive than cold knife conisation and may be associated with the removal of smaller amounts of tissue, resulting in a higher risk of positive cone margins than in the past [71]. The increased surveillance of patients who have this characteristic may lead to an overestimate of the risk of VC [40], especially in the first years after treatment. On the other hand, however, CIN3 is a screen-detected lesion, so that patients are likely to have an active health behaviour and to regularly attend follow-up visits. This moderates the risk of progression of their vulvar disease to an invasive VC. The balance between these two influences seems to be in favour of an increased detection of VC early after CIN3 treatment [47], with an inverse relationship between the length of follow-up and the risk of disease.

The potential implications of surveillance of patients treated for CIN3 were illustrated by a Finnish hospital-based cohort study in which, paradoxically, the risk of cervical cancer was lower for patients treated for CIN3 than for those treated for CIN1 and CIN2 [56]. According to the authors, the most likely explanation was a greater intensity of post-treatment surveillance for the former.

Bjørge et al. noted that women treated for CIN3 were at increased risk of those cancer types most strongly associated with cigarette smoking (in particular, lung cancer and bladder cancer as well as VC) and emphasized the role of cigarette smoking as a shared risk factor [25]. The model of vulvar carcinogenesis postulated by zur Hausen for integrating HPV infection and smoking is briefly illustrated below (see Section 4.3.8).

#### 4.3.4. VLS

A diagnosis of VLS is often considered an established risk factor for VC and VSCC. The epidemiologic evidence, however, relies on no more than two studies [34,48]. According to a recent hypothesis, the true precursor of VC would be dVIN, which frequently coexists with VLS [93,94]. The 10-year cumulative incidence of VSCC is 18.8% among VLS patients who have concurrent dVIN at baseline vs. 2.8% among those who are free of dVIN [95]. However, a measure of the risk of VC for women with dVIN alone is not yet available.

In the Finnish nationwide study [48], addressing VSCC, the risk of cervical cancer among women with VLS was dramatically decreased (SIR, 0.00; 95% CI, 0.00–0.70). The authors interpreted this unexpected finding as consistent with a decreased exposure to HPV (caused by patients’ sexual impairment), a lower rate of smoking (suggested by the low risk of lung cancer among Finnish patients), and more intensive cervical screening. As stated above, the prevalence of screening introduces a potential bias into the estimates of the excess risk of cervical disease [48,65], and may also influence the progression to VC.

#### 4.3.5. SLE

All of the five cohort studies of patients with SLE yielded positive results [22,32,38,62,69]. It has previously been suggested that the elevated risk of VC in women with SLE may depend on a common inflammatory mechanism [16,96]. The chronic inflammation may cause continuous apoptosis, tissue injury, wound healing processes and changes in cancer-associated genes [17]. Injuries that do not heal require a constant renewal of cells, which increases the likelihood of neoplastic transformation [97]. However, there is a limited literature linking multiple chronic inflammatory conditions to VC. One of the articles selected for this review, for example, reported a significant association between prior histories of leucoplakia and inflammation with the risk of VC but without a formal measure [58]. As a consequence, the current view is that the higher incidence of VC should be primarily ascribed to an increased susceptibility to HPV infection [16]. The risk of infection can further increase due to the immunosuppressive therapy [98] or an impairment of the viral clearance [22,38]. The literature, however, suggests that SLE could be linked to HPV by a bidirectional relationship. The immunosuppressive drugs can increase the risk of HPV infection but, conversely, the immune responses following HPV infection may cross-react with proteins that, when altered, are associated with the risk of SLE [99].

#### 4.3.6. Menstrual and Reproductive Factors, Oral Contraceptive and Menopausal Hormone Use

Five studies [30,33,58,68,79] have investigated the effects of many menstrual and reproductive factors, oral contraceptive use, and menopausal hormone replacement therapy. The results have been largely negative. In a case-control study, Sherman et al. found only a weakly significant increase in the risk of VSCC for women reporting a history of abortion [79]. In the Million Women study, a significant 50% increased risk of VSCC was observed among women aged < 50 years at menopause or oophorectomy who never used hormone therapy [33].

The latter observation might be interpreted as suggesting that early or premature menopause and the resulting decrease in oestrogen exposure over lifetime might be a risk factor for VC. Evidence from mouse models suggests that oestrogen exposure has an inhibitory role in the growth of squamous cell tumours. On the other hand, oestrogen is known to promote cancer in several oestrogen-responsive tissues, including the cervix. Thus, it appears that oestrogen may have both tumorigenic and antitumour properties, depending on the type of tissue targeted and on the presence of receptors [100]. Further basic science research is needed to elucidate the role of oestrogen in the development of VC.

#### 4.3.7. Metabolic Syndrome, Diabetes, BMI, Food Items

Metabolic syndrome is a cluster of metabolic abnormalities that include hypertension, central obesity, insulin resistance, and atherogenic dyslipidemia. Five studies have consistently demonstrated that metabolic syndrome and BMI are associated with an increased risk of VC [30,33,64,68] and VSCC [79].

The components of the metabolic syndrome, too, have one or more plausible relationships with vulvar carcinogenesis. The association between hypertriglyceridemia and VC risk may depend on the fact that hypertriglyceridemia causes frequent infections and inflammation, which may include HPV infection [64,101]. The effect of high blood glucose levels may be due to the association with vulvar dystrophies and chronic dermatitis [64]. Obesity and elevated concentrations of blood glucose and triglycerides contribute to the development of hyperinsulinemia and the maintenance of low-grade systemic inflammatory milieu [64,101]. Regarding diabetes, however, there have also been negative studies, in particular those of Coffey et al. [33] and Brinton et al. [30].

With respect to BMI, it correlates strongly with endogenous sex-steroid concentrations, and the alterations in sex-steroid hormones associated with increasing body weight are among the potential mechanisms underlying the observed association with VC [64]. Obesity is also associated with hyperinsulinemia [64,101].

The effects related to single food items have been insufficiently investigated. One study associated a low consumption of green vegetables and a high consumption of red meat with the risk of VC [68]. There are some more studies regarding alcohol consumption, but their results have been inconsistent [30,33,61,68,87].

#### 4.3.8. Smoking

Six out of the eight studies reviewed have reported positive findings on the association between smoking and the risk of VC [29,30,33,58,59,68] and VSCC [36,61]. Smoking has a preeminent role in the HPV-related carcinogenesis, including vulvar carcinogenesis. The hypothesis that smoking would participate in this process through local immunosuppression and/or direct carcinogenetic effects [14] has been reconsidered. HPV-related carcinogenesis is currently thought to be a multistage process that requires cofactors to cause the malignant transformation. Zur Hausen proposed a synergistic model in which HPV causes cell hyperplasia and, subsequently, a carcinogen induces cell transformation [102]. Smoking is hypothesised to be this cofactor. There have also been in vitro studies suggesting that the byproducts of smoke can transform HPV-immortalized cell lines [103]. In addition, smoking may act through the inhibition of apoptosis, an effect that is attributed to nicotine. In brief, the combination of smoking and HPV abrogates the control on two components of cell kinetics: proliferation and programmed cell death [59]. The epidemiologic data are well in keeping with these hypotheses. In the study by Daling et al., the presence of both genital warts and smoking was associated with a risk of VSCC largely greater than one would see if the effects of the two exposures were additive [36]. Compared with nonsmokers free of genital warts, nonsmokers with genital warts had an OR of 7.8 and smokers free of genital warts an OR of 4.2. When both exposures were present, the OR rose to 51.3.

#### 4.3.9. HIV-AIDS

Even though with a substantial variability in risk estimate, all of the eight studies on the association of HIV-AIDS with VC [44,50,51,63,66], V/VC [80,83] and V/VSCC [46] have reported positive findings.

The underlying mechanisms are complex. In part, the excess risk depends on the higher prevalence of well-established cancer risk factors including, for example, smoking and alcohol use as well HPV coinfection. HIV infection and the associated immunodeficiency, however, contribute to the risk increase with a reduced immune surveillance against transformed cells and HPV itself [80]. In general, the increased risk of cancer among HIV-infected people is accounted for by infection-related cancers, including HPV-related cancers.

Despite this evidence, the relationship between HIV and HPV remains a stimulating area of research, especially if investigated in high-incidence populations [20]. In a case-control study from Rwanda, for example, the authors found a stronger association of HIV infection with the risk of VC compared with the risk of cervical cancer [63]. This was interpreted as suggesting a more direct role of immunosuppression in vulvar carcinogenesis.

Another intriguing observation regarding the interplay between race/ethnicity and HIV infection has been reported from the US. It is known that Hispanic Americans are disproportionately affected by HIV and by infection-related cancers. In a cohort study of HIV-infected people, Hispanic women were demonstrated to be at higher risk of cervical cancer than non-Hispanic whites but at lower risk of VC as compared with HIV-infected non-Hispanic whites as well as non-Hispanic Blacks [66]. An opposite pattern, however, was observed in a study of American women with AIDS, where those of Hispanic origin showed a considerably higher risk of V/VSCC than the general population [46]. The reasons for these conflicting observations need to be clarified. Although methodological issues, especially in the selection of the reference population, might account for the above findings, further research on other biological and social contributing factors has been advocated [66].

In our design, we considered not relevant to the study the effects of exposure to drugs, including the effects of antiretroviral therapy. Aside from this, it should be considered that the advent of antiretroviral therapy has reduced the excess risk of cancer among HIV-infected people.

#### 4.3.10. Solid-Organ Transplantation

We identified 12 studies on the association between solid-organ transplantation (renal transplantation in most instances) and VC [21,41,43,60,77,84,86,88], VSCC [73] and V/VC [23,81,82]. All reported a risk increase, which was generally substantial but varied to a great extent. In addition to differences in the design of studies, potential explanations do probably include differences in the age at transplantation, in the immunosuppressive regimen, and in the intensity of cancer screening practices [86]. It must be considered that medical surveillance of these patients is heightened both before and after transplantation.

One of the lowest SIRs observed among renal transplant recipients, 5.81, was reported by Skov Dalgaard et al. [82]. The study population included both transplant recipients and patients undergoing chronic dialysis. Although justified from a clinical perspective, this approach pooled patients who have very different levels of risk. Fairley et al. reported distinct estimates showing a 10-fold larger excess risk for transplanted patients [43]. Moreover, the increase associated with chronic dialysis was not significant. Skov Dalgaard et al. themselves confirmed a significant difference between the two subpopulations. At present, the evidence for an association of chronic dialysis with the risk of VC is poor.

Several hypotheses have been raised to explain the excess of cancer among solid-organ transplant recipients. The mechanisms that have been most commonly invoked include immune modulation and infection with HPV and, possibly, with other oncogenic viruses (for example, Epstein–Barr virus and HIV) and with Helicobacter pylori [21]. The pattern of excess risk at multiple sites that is seen among transplant recipients, however, is very complex and challenges our understanding of the oncogenic infections potentially activated by immunologic alterations [21]. By implication, this remains an area for further research. Special attention should be paid to paediatric organ transplantation, because patients may suffer from a more pronounced risk increase [81] due to improved life expectancy and prolonged exposure [21]. To the best of our knowledge, also, it remains to be fully confirmed that only older maintenance immune suppressive drug regimens, specifically azathioprine, are associated with an increased risk of VC [60].

#### 4.3.11. Breast Implants

Cosmetic breast implant surgery is increasingly popular and, albeit anecdotal, the associated increase in the risk of some cancer types deserves attention. The excess of V/VC observed in the study by Brinton et al., however, should be cautiously interpreted, because it seems more easily attributable to reproductive and lifestyle risk factors [28]. In the same study, an excess risk of cervical cancer was also observed. When comparing women with cosmetic breast implants with other plastic surgery patients, the risk increase was no longer significant. This would suggest that women undergoing plastic surgery are exposed to risk factors other than silicone. It is known, for example, that women with breast implants are more likely to drink a greater average number of alcoholic drinks per week, to be younger at first pregnancy, to have ever used oral contraceptives, and to have had a greater lifetime number of sexual partners [104]. This poses the question of insufficient adjustment for confounders that affects some studies.

#### 4.3.12. Uncommon Risk Factors

Albeit unconfirmed, the recent study by D’Arcy et al. reporting an inverse association between the presence of allergic rhinitis and V/VC is intriguing [35]. When the allergic reaction occurs, mast cells release mediators, particularly cytokines, which may promote an immune response against precancerous cells. It is increasingly clear that the immune system, in addition to facilitating tumour growth by providing a favourable tumour microenvironment, can also have a tumour-suppressive function with the elimination of nascent transformed tumour cells [105]. The distinction between tumour-promoting inflammation and tumour-suppressive immunity, however, is not completely understood yet given the dual role of some cytokines and other molecules [105]. The inverse relationship between the presence of allergic rhinitis and the risk of V/VC has also been explained with the hypothesis that the drugs used to treat allergic rhinitis have chemopreventive properties [35].

### 4.4. Methodological Issues

Several methodological issues of this study need to be clarified. First, we did not present detailed analyses for individual risk factors, nor we did assess in a formal fashion the quality of articles and the between-study heterogeneity. Our aim was to describe—for the first time, to our knowledge—a general and comprehensive framework of past and current state of epidemiologic research on this condition, with attention to time trends and emerging research lines, assuming that this may provide insights for future directions. Consequently, our selection encompassed all potential and putative determinants of the risk of VC reported from studies with a few simple design requirements. A meta-analytic approach would not be appropriate for this design nor feasible in a single report.

This study was not aimed at reviewing the risk factors for preinvasive vulvar disease. For several exposures, however, we incidentally noted different patterns of risk (presence/absence and strength) between preinvasive vulvar disease (whatever its definition) and invasive VC. In particular, several factors had a stronger influence on the risk of preinvasive conditions. Hildesheim et al., for example, demonstrated that HPV-16 seropositivity was associated with a higher risk of VIN3 than invasive VC [52]. Studies by Brinton et al. demonstrated that the number of births and the use of oral contraceptive and menopausal hormone therapy had a significant effect on the risk of VIN3 but not of invasive VC [30], and that the number of sexual partners and current smoking were stronger risk factors for in situ disease [29]. In a study by Madeleine et al., transplant recipients experienced a threefold larger increase in the risk of in situ VC compared with invasive cancer [60]. A study of women with cosmetic breast implants, too, showed a significantly increased risk of in situ VC but not of the invasive counterpart [37]. This apparently univocal pattern is particularly provocative, and warrants an explanation. In any case, it is clear that extrapolating the epidemiology of preinvasive to invasive disease and pooling the two entities in risk estimates [29] may be strongly misleading.

In several studies reviewed here, the risk of VC and that of vaginal cancer were cumulated. We decided to include this literature in our work for two reasons. First, this approach might be justified for those studies that have used cancer registry data affected by some degree of mutual misclassification of vulvar and vaginal cancers. This problem is a common concern to cancer registrars [106]. Second, we believe that it is important to objectively illustrate the type of evidence currently available, including its limitations. Aside from this, however, the risk of vulvar and vaginal cancer associated with certain exposures may differ considerably and in opposite directions. For women diagnosed with CIN3, for example, the risk of vaginal cancer exceeds constantly the risk of VC [39,40,42,55,56,67,71] reflecting the close proximity of the vaginal epithelium to cervical abnormalities. Conversely, a history of anogenital warts [26], VLS [48], and solid organ transplantation [21] increases the risk of VC (or VSCC) to a greater extent. Thus, it appears that pooled risk estimates may be fallacious for the planning of follow-up strategies. Last, combining the two entities is in contradiction with the fact that, while vaginal carcinomas are mainly HPV-related, most of VCs derives from an HPV-independent pathway [9] and has different precursor lesions and different clinical and pathological features [107]. VC often arises in a context of vulvar dermatosis, although the role of the inflammation is still under investigation, and particularly in women with a history of VLS. VLS is a frequent disease of the vulva but is rarely located in the vagina and does not have a demonstrated link to vaginal malignancies [108].

The two pathways in the development of VC, one related to and the other independent of HPV, will be discussed below (see Section 4.5). It is important to note here that apparently no consideration was given in the epidemiologic literature to the fact that the two carcinogenic models are not mutually exclusive. Women with VLS can be infected with HPV and HPV-infected women can develop VLS. Unfortunately, the histologic criteria alone are not sufficient to differentiate between HPV-dependent and HPV-independent VSCC [107], and a HPV-positive tumour can arise in precursor lesions simulating dVIN [109]. The two pathways may have similar steps [110]. This is relevant for the design of future studies.

Nearly 80% of studies were unable to separate VC according to the histologic type and, in particular, to restrict the analysis to VSCC. For example, the endpoint of all of the 10 studies of women with a history of CIN1-3 was the risk of VC or V/VC. Pooling the histologic types together may be due to limitations in availability and quality of data, and is encouraged by statistical power considerations and by the fact that the greater part of VCs, approximately 90% [78,111], have a squamous cell carcinoma histology. The implications should be viewed from two different angles. On the one hand, the strength of an association may differ between VSCC and the other types. For example, Coffey et al. observed that obese women (BMI >30.0) had little or no increased risk for non-squamous VC, including basal cell, glandular, and melanocytic tumours [33]. If pooled with VSCC, the latter types may theoretically introduce a bias towards the null hypothesis of no association. The inclusion of non-squamous VCs is particularly incoherent in those studies addressing the exposure to a risk factor –for example, an inflammatory condition– that is expected to be associated with VSCC alone. On the other hand, the low proportion of non-squamous VCs limits their biasing potential, although it remains necessary that future research refines its methodology.

In cohort studies, different approaches were used to deal with women diagnosed with VC early after recruitment. For example, women diagnosed within one year were retained in the study of Halonen et al. (women with VLS) [48] and Mellemkjaer et al. [62] (women with SLE), while being excluded from those of Pan et al. (women with CIN3) [67] and Weiderpass et al. (women with a hospital discharge diagnosis of alcoholism) [87]. In a Canadian cohort study of renal transplant recipients, only patients diagnosed with cancer in the 30-day period immediately after transplantation were excluded [86]. In a US cohort study of HIV-infected people, Hernández-Ramírez et al. removed from analysis the first three months of follow-up [50]. In a cohort of women with CIN2-3, Gaudet et al. excluded those patients diagnosed with VC within six months [47]. Parikh-Patel et al. (women with SLE) [69] and Corazza et al. (women with VLS) [34] used the same criterion, but the latter research group still found a huge excess incidence of VC between six and 36 months of follow-up. Others performed a sensitivity analysis by examining the extent to which the results were affected by the exclusion of the first year of follow-up [39]. These different approaches may considerably influence, in particular, the results of follow-up studies of patients with high-grade CIN, because their risk of VC shows an early peak [39,47]. According to the data reported by Halonen et al., a higher risk of VSCC is also observed during the first year of follow-up of patients with VLS [48]. The authors raised the hypothesis that patients with close diagnosis of VLS and subsequent cancer do probably seek medical care because of symptoms of cancer instead of symptoms of LS. If so, this would introduce a bias in the association between the two conditions.

We focused on the risk of first cancer alone. Failure to distinguish between the risk of a first primary cancer from that of a second cancer would result in an underestimate of incidence. The reasons are illustrated in the literature [51].

Our classification of the type of population studied was approximated in some instances. For example, the cohort reported by Birkeland et al. [23] included all patients from all transplantation centres in the Nordic countries. We classified this study as a population-based one, although the source of data was formally different from a standard population-based organ transplantation registry.

Finally, four articles were impossible to retrieve in full text [112,113,114,115]. Based on information in the abstracts, we believe that these articles would not change the overall picture provided by our study.

### 4.5. Summary of Evidence

As mentioned in the Introduction section, it is commonly assumed that there exist two main models of vulvar carcinogenesis [8,107]. The first is centred on HPV infection and HPV-related diseases coupled with different defects of the immune function. VSCC accounts for about 90% of VCs [111] and one-third of VSCCs are HPV-positive [9], with HPV type 16 being involved in 75% of cases. HPV-positive VSCC follows the HPV-dependent carcinogenic pathway through high-grade squamous intraepithelial lesions. HPV-associated VSCC arises at a younger age and more often shows warty and basaloid features. Inactivation of tumour suppression genes (p53 and retinoblastoma), due to E6–E7 viral proteins, and the very frequent p16 immunoreactivity [8,116] are the hallmarks of the HPV-dependent pathway.

Several of the risk factors proposed in the literature can be incorporated into this model. SLE is accompanied by an increased susceptibility to HPV infection, and it is hypothesised that the two conditions are linked by a bidirectional relationship [98,99]. Solid-organ transplantation and HIV-AIDS are associated with immunosuppression and enhancement, in particular, of the virus-related carcinogenesis. Hypertriglyceridemia causes frequent infections, which may include HPV infections [64,101]. Allergies exert a protective effect from the risk of VC by promoting the immune response, including the response to precancerous cells [35]. Finally, smoking is the cofactor that induces cell transformation in a background of epithelial hyperplasia caused by the HPV infection [102]. Our work shows that, for most of these risk factors, in particular HPV infection, history of CIN3, history of SLE, smoking, HIV/AIDS, and solid-organ transplantation, there is increasing epidemiologic evidence for a role in vulvar carcinogenesis. To some extent, the two studies suggesting a relationship between a history of abnormal Pap smear and the risk of VC may be considered to add support to the HPV-dependent model of vulvar carcinogenesis [78,85]. However, we have classified these studies separately from those dealing with HPV infection and CIN1-3 because a cytologically reported cervical abnormality often predicts benign inflammatory or reactive changes. This is the likely reason why a third study of women with previous abnormal Pap smears yielded negative results as to the risk of VC [29].

The second model of vulvar carcinogenesis, which is centred on the association between VLS and the risk of VC, is considered the most common one, accounting for up to 70% VSCC cases [9]. However, it clearly appears from our findings that this model has been much less investigated than the HPV-dependent model. We have identified only two eligible studies dedicated to exploring it in a formal fashion [34,48]. VLS is a T-cell mediated inflammatory dermatosis involving vulvar labia majora and minora, clitoris, posterior fourchette and perineum, with unknown aetiology and multifocal and sometimes symmetrical lesions. Those VSCCs that follow this pathway arise usually in post-menopausal women and show a keratinizing pattern. It must be considered, however, that a clear progression from VLS to VSCC has not been identified yet, although chronic inflammation is likely to involve molecular alterations and genetic mutations.

Currently, one accredited hypothesis is that dVIN is the true HPV-independent precursor of VC [93,94]. In fact, this relationship has never been studied with a formal epidemiologic approach and still awaits a confirmation. Some researchers evaluated cohorts of patients with dVIN in order to determine the cumulative rate of progression to VC but without an unaffected control population. Bleeker et al. compared VLS patients with concurrent dVIN and VLS patients free of dVIN [95]. The cohort study by Thuijs et al. compared patients with dVIN vs. patients with vulvar high-grade squamous intraepithelial lesion [117]. Others have simply reported the prevalence of VC among patients with dVIN [118,119]. In summary, we can confirm the opinion of Bigby et al. that the role of dVIN in the HPV-independent pathway of vulvar carcinogenesis is still based on largely circumstantial observations [120].

The huge difference in the amount of literature supporting the two carcinogenic models is, at least in part, easy to explain. The epidemiologic research on the HPV-dependent pathway has been greatly facilitated by the availability of many large, population-based, often nation-wide registries of persons with a history of CIN3, SLE, HIV/AIDS, and solid-organ transplantation. This is not (or not yet) the case for patients with VLS and dVIN. The creation of multicentre hospital registries (ideally international registries), in parallel with the adoption of standard definitions and uniform criteria for differential diagnosis, may be the only solution to overcome this problem [121].

### 4.6. Previous Systematic Reviews and Meta-Analyses

Some of the above risk factors have previously been the subject of four meta-analyses and three systematic reviews. Kalliala et al. investigated the effect of a history of CIN3 as reported by seven cohort studies with at least five years of follow-up and cancer registry-based ascertainment of incident cancer [19]. The pooled RR was 3.34 (95% CI, 2.39–4.67). There was a significant between-study heterogeneity, which was reduced by the inclusion of European studies alone.

Three meta-analyses evaluated the results of studies on patients with SLE. Cao et al. considered four cohort studies published between 1997 and 2011. The pooled RR was 3.67 (95% CI, 2.80–4.81) [16]. The degree of heterogeneity between studies was moderate. Mao et al. evaluated the cohort, case-control and observational studies published through 2015 [17]. They reported a pooled risk ratio of V/VC of 4.04 (95% CI, 3.00–5.43). In a third meta-analysis of cohort studies, Song et al. considered the studies published between 2003 and 2018 [18]. The SIR for V/VC was 3.48 (95% CI, 2.69–4.50). No significant heterogeneity between studies was found.

Of the three eligible systematic reviews, the one reported by Moore et al. considered the articles published through 1998 and aimed at investigating the interplay between HPV and smoking [14]. They concluded that sufficient evidence already existed that the increase in the risk of VC is particularly strong among women who are both current smokers and HPV-16 seropositive. In 2010, Hjartåker et al. reviewed the available evidence for an association between alcohol consumption and VC [15]. They stated that no conclusion could be drawn. Lekoane et al. focused on the risk of HPV-related cancers specifically for HIV-infected sub-Saharan women [20]. They made no conclusions from the sparse literature available.

## 5. Conclusions

The cumulative body of epidemiologic work carried out in the last years, especially in the past decade, has provided us with interesting insights about the multifaceted aetiology of VC. Recent advances have yielded further evidence in support of the carcinogenic model centred on HPV infection with different defects of the immune function. Conversely, the model centred on the role of VLS and dVIN has continued to be epidemiologically understudied and awaits still a confirmation. More research on the association between these two conditions and VC is a priority.

## Figures and Tables

**Table 1:** Selected results from descriptive studies on vulvar cancer age-standardised (world standard population) incidence rates per 100,000 women in different time periods and countries.

	First Author: Kang [12]				First Author: Bray [6]
	1988–1992	1993–1997	1998–2002	2003–2007	2008–2012
Iceland	1.27	1.33	1.02	0.92	1.50
Sweden *	1.34	1.42	1.48	1.44	NA
Denmark	1.34	1.5	1.49	1.68	1.70
Ireland	1.07	1.02	1.18	1.30	1.40
United Kingdom †	1.43	1.54	1.62	1.68	1.90
The Netherlands	1.31	1.36	1.44	1.66	2.10
Saarland (Germany)	1.49	1.18	1.38	4.08	5.70
France	0.91	0.90	0.96	0.92	1.10
Switzerland	1.11	1.28	1.05	1.27	1.30
Canada	1.35	1.30	1.41	1.40	1.70
SEER 9	1.43	1.35	1.49	1.46	1.50
Japan	0.24	0.24	0.22	0.26	0.40
Australia	1.16	1.36	1.25	1.40	1.60
	**First author, Bray [10]**				
	**1990**	**1995**	**2000**	**2005**	
Norway	1.25	1.42	1.35	1.55	1.60
Slovakia	1.17	1.30	1.40	1.30	1.50
Cali (Colombia)	0.81	1.21	0.92	0.91	0.80
Chennai (India)	0.59	0.64	0.6	0.54	0.40
Shangai (China)	0.25	0.16	0.37	0.26	0.40

* Sweden was not included in *Cancer Incidence in Five Continents,* vol. XI.; † The Oxford Cancer Registry was not included in *Cancer Incidence in Five Continents*, vol. XI.

**Table 2:** Summary of cohort and case-control studies on all known and putative risk factors for vulvar cancer published between 1980 and 2020. Part 1.

Risk Factor	First Author * [Ref.]	Year	Country	Design	Representativeness	Exposed	Cases	Controls	Age †
HPV-16 seropositivity (>0.100) ‡	Bjørge [24]	1997	Norway	CC	Study nested in a nationwide population-based cohort	NA	25	73	Median, 45
HPV16-seropositivity	Hildesheim [52]	1997	US	CC	Not specifiable	NA	77	63	Range, 20–79
HPV16-seropositivity	Madeleine [59]	1997	US	CC	3-county, cancer-registry-based case series	NA	110	1403	52% ≥60
HPV6-seropositivity	Madeleine [59]	1997	US	CC	3-county, cancer-registry-based case series	NA	110	1403	52% ≥60
HPV18-seropositivity	Madeleine [59]	1997	US	CC	3-county, cancer-registry-based case series	NA	110	1403	52% ≥60
HPV2-seropositivity	Madeleine [59]	1997	US	CC	3-county, cancer-registry-based case series	NA	110	1403	52% ≥60
HPV16-L1 seropositivity	Kreimer [57]	2015	US	CC	Study nested in an international European cohort	NA	67	658	Median, 65
HPV16-E6 seropositivity	Kreimer [57]	2015	US	CC	Study nested in an international European cohort	NA	67	658	Median, 65
HPV16-E7 seropositivity	Kreimer [57]	2015	US	CC	Study nested in an international European cohort	NA	67	658	Median, 65
HPV16-E1 seropositivity	Kreimer [57]	2015	US	CC	Study nested in an international European cohort	NA	67	658	Median, 65
HPV16-E2 seropositivity	Kreimer [57]	2015	US	CC	Study nested in an international European cohort	NA	67	658	Median, 65
HPV16-E4 seropositivity	Kreimer [57]	2015	US	CC	Study nested in an international European cohort	NA	67	658	Median, 65
Fam. cluster. of HPV-rel. cancers	Hussain [53]	2008	Sweden	C	Nationwide, population-based cohort	3,625,784	107	NA	Range, 0–72
Fam. cluster. of HPV-rel. cancers	Hussain [53]	2008	Sweden	C	Nationwide, population-based cohort	3,625,784	83	NA	Range, 0–72
Fam. cluster. of HPV-rel. cancers	Zhang [89]	2019	Germany	C	Nationwide, population-based cohort	NR	7	NA	Median, 59
Fam. cluster. of HPV-rel. cancers	Zhang [89]	2019	Germany	C	Nationwide, population-based cohort	NR	17	NA	Median, 59
Genital warts	Brinton [29]	1990	US	CC	Multicentre hospital-based case series	NA	209	113	Mean, 54
Genital warts	Sherman [78]	1991	US	CC	3-county, cancer-registry-based case series	NA	53	466	Range, 18–79
Genital warts	Blomberg [26]	2012	Denmark	C	Nationwide, population-based cohort	33,422	74	NA	Median, 23
Anogenital warts	Madsen [61]	2008	Denmark	CC	Nationwide, cancer-registry-based series	NA	116	518	Median, 63
Anogenital warts in the partner	Madsen [61]	2008	Denmark	CC	Nationwide, cancer-registry-based series	NA	116	518	Median, 63
Condylomata acuminata	Friis [45]	1997	Denmark	C	Nationwide, population-based cohort	9552	11	NA	Median, 24
Condylomata acuminata	Nordenvall [65]	2006	Sweden	C	Nationwide, population-based cohort	9286	13	NA	Median, 23
Coital experience	Mabuchi [58]	1985	US	CC	Multicentre hospital-based case series	NA	149	149	81% ≥50
Age at first coitus	Mabuchi [58]	1985	US	CC	Multicentre hospital-based case series	NA	149	149	81% ≥50
Age at first coitus	Sherman [78]	1991	US	CC	3-county, cancer-registry-based case series	NA	53	466	18–79
No. of sexual partners	Brinton [29]	1990	US	CC	Multicentre hospital-based case series	NA	209	112	Mean, 54
No. of sexual partners	Sherman [78]	1991	US	CC	3-county, cancer-registry-based case series	NA	53	466	Range, 18–79
No. of sexual partners	Parazzini [68]	1995	Italy	CC	Hospital-based case series	NA	125	541	Median, 63
No. of sexual partners	Hildesheim [52]	1997	US	CC	Not specifiable	NA	77	63	Range, 20–79
No. of sexual partners	Madsen [61]	2008	Denmark	CC	Nationwide, cancer-registry-based series	NA	116	518	Median, 63
No. of marriages	Mabuchi [58]	1985	US	CC	Multicentre hospital-based case series	NA	149	149	81% ≥50
No. of marriages	Sherman [78]	1991	US	CC	3-county, cancer-registry-based case series	NA	53	466	Range, 18–79
Age at first marriage	Mabuchi [58]	1985	US	CC	Multicentre hospital-based case series	NA	149	149	81% ≥50
Anal intercourse	Madsen [61]	2008	Denmark	CC	Nationwide, cancer-registry-based series	NA	116	518	Median, 63
Genital washing b/a intercourse	Madsen [61]	2008	Denmark	CC	Nationwide, cancer-registry-based series	NA	116	518	Median, 63
Partner’s marital status	Madsen [61]	2008	Denmark	CC	Nationwide, cancer-registry-based series	NA	116	518	Median, 63
Partner’s no. of sexual partners	Madsen [61]	2008	Denmark	CC	Nationwide, cancer-registry-based series	NA	116	518	Median, 63
**Risk Factor**	**[Ref.]**	**Comparison**	**Disease**	**Measure**	**Result (95% CI)**	**Adjustment variables**			
HPV-16 seropositivity (>0.100) *	[24]	Exposure vs. no exposure	V/VC	OR	5.5 (1.5–25)	Age at sampling, county, storage time			
HPV16-seropositivity	[52]	Exposure vs. no exposure	VC	OR	2.9 (0.94–8.7)	Age, EDU, smoking, years of OC, no. of sex. partners, HSV, Chlamydia			
HPV16-seropositivity	[59]	Exposure vs. no exposure	VSCC	OR	2.8 (1.7–4.7)	Age, EDU, smoking, BMI			
HPV6-seropositivity	[59]	Exposure vs. no exposure	VSCC	OR	1.2 (0.7–2.3)	Age, EDU, smoking, BMI			
HPV18-seropositivity	[59]	Exposure vs. no exposure	VSCC	OR	1.2 (0.5–2.7)	Age, EDU, smoking, BMI			
HPV2-seropositivity	[59]	Exposure vs. no exposure	VSCC	OR	1.5 (0.9–2.6)	Age, EDU, smoking, BMI			
HPV16-L1 seropositivity	[57]	Exposure vs. no exposure	VC	OR	3.4 (1.8–6.4)	Age, country, smoking			
HPV16-E6 seropositivity	[57]	Exposure vs. no exposure	VC	OR	4.0 (0.4–46.0)	Age, country, smoking			
HPV16-E7 seropositivity	[57]	Exposure vs. no exposure	VC	OR	1.1 (0.4–2.9)	Age, country, smoking			
HPV16-E1 seropositivity	[57]	Exposure vs. no exposure	VC	OR	0.9 (0.2–3.0)	Age, country, smoking			
HPV16-E2 seropositivity	[57]	Exposure vs. no exposure	VC	OR	1.5 (0.5–4.3)	Age, country, smoking			
HPV16-E4 seropositivity	[57]	Exposure vs. no exposure	VC	OR	1.0 (0.5–1.9)	Age, country, smoking			
Fam. cluster. of HPV-rel. cancers	[53]	See footnote §,	VSCC	SIR	1.80 (1.48–2.18)	Age, period, area of residence, SES			
Fam. cluster. of HPV-rel. cancers	[53]	See footnote ¶	VSCC	SIR	1.76 (1.40–2.18)	Age, period, area of residence, SES			
Fam. cluster. of HPV-rel. cancers	[89]	See footnote **	V/VC	SIR	2.38 (1.14–5.01)	Age, period, area of residence, SES			
Fam. cluster. of HPV-rel. cancers	[89]	See footnote ††	V/VC	SIR	2.72 (1.69–4.39)	Age, period, area of residence, SES			
Genital warts	[29]	Exposure vs. no exposure	VC	RR	14.55 (1.7–125.6)	Age, smoking, no. of sexual partners, previous abnormal Pap smear			
Genital warts	[78]	Exposure vs. no exposure	VSCC	OR	17.3 (6.3–47.2)	Age, period, EDU, smoking, no. of sexual partners			
Genital warts	[26]	Exposed vs. general population	VC	SIR	14.8 (11.7–18.6)	Age, year			
Anogenital warts	[61]	Exposure vs. no exposure	VSCC	OR	5.77 (3.08–10.8)	Age, EDU, smoking, alcohol, marital status			
Anogenital warts in the partner	[61]	Exposure vs. no exposure	VSCC	OR	2.04 (0.56–7.48)	Age, EDU, smoking, alcohol, marital status, anogenital warts			
Condylomata acuminata	[45]	Exposed vs. general population	VC	SIR	40.1 (20.0–71.7)	Age, period			
Condylomata acuminata	[65]	Exposed vs. general population	VC	SIR	10.2 (5.4–17.4)	Age, year			
Coital experience	[58]	Never vs. ever	VC	OR	1.53 (NS)	NR			
Age at first coitus	[58]	≥26 vs. <16	VC	OR	1.19 (NS)	NR			
Age at first coitus	[78]	≥21 vs. ≤16	VSCC	OR	1.1 (0.4–3.2)	Age			
No. of sexual partners	[29]	≥10 vs. 0–1	VC	RR	0.83 (0.3–2.5)	Age, smoking, genital warts, previous abnormal Pap smear			
No. of sexual partners	[78]	≥15 vs. 0–1	VSCC	OR	8.2 (2.3–29.1)	Age			
No. of sexual partners	[68]	≥3 vs. 0–1	VC	OR	1.9 (0.8–4.1)	Age, EDU, BMI			
No. of sexual partners	[52]	≥3 vs. 0–1	VC	OR	3.4 (1.5–7.7)	Age, EDU, age started smoking, years of OC, HSV, chlamydia			
No. of sexual partners	[61]	≥10 vs. 2–4	VSCC	OR	0.71 (0.31–1.65)	Age, EDU, smoking, alcohol, marital status, anogenital warts			
No. of marriages	[58]	≥2 vs. 0	VC	OR	0.94 (NS)	NR			
No. of marriages	[78]	≥3 vs. 1	VSCC	OR	4.6 (2.0–10.6)	Age			
Age at first marriage	[58]	≥30 vs. <20	VC	OR	3.29 (NR, *p* < 0.05)	NR			
Anal intercourse	[61]	Ever vs. never	VSCC	OR	0.67 (0.31–1.44)	Age, EDU, smoking, alcohol, marital status, anogenital warts			
Genital washing b/a intercourse	[61]	10/10 times vs. 6–9/10	VSCC	OR	0.82 (0.45–1.48)	Age, EDU, smoking, alcohol, marital status, anogenital warts			
Partner’s marital status	[61]	Unmarried vs. married	VSCC	OR	0.20 (0.04–0.92)	Age, EDU, smoking, alcohol, marital status, anogenital warts			
Partner’s no. of sexual partners	[61]	0 vs. ≥3	VSCC	OR	0.66 (0.31–1.41)	Age, EDU, smoking, consumption, marital status, anogenital warts			

BMI = body mass index; C = cohort; CC = case-control; EDU = education; Fam. clust. of HPV-rel. cancers = familial clustering of HPV-related cancers; HPV = human papillomavirus; HSV = herpes simplex virus; NA = not applicable; NR = not reported; NS = not significant; OC = oral contraceptive; OR = odds ratio; ref. = reference; RR = relative risk; SES = socio-economic status; sex. = sexual; SIR = standardised incidence ratio; V/VC = vulvar/vaginal cancer; VC = vulvar cancer; vs. = versus; VSCC = vulvar squamous cell carcinoma; US = United States. * In the case of international authorship, the country of the first author is indicated. † The best information made available in the article is indicated. In general, age refers to the age of cases at diagnosis in case-control studies and the age at entry into cohort studies. If possible, age is expressed in completed years. ‡ The cut-off point is expressed in absorbance units. § Offspring with a sister with cervical squamous carcinoma vs. general population. ¶ Offspring with the mother with cervical squamous carcinoma vs. general population. ** Offspring with a family member with anal cancer vs. general population. †† Offspring with a family member with V/VC vs. general population.

**Table 3:** Summary of cohort and case-control studies on all known and putative risk factors for vulvar cancer published between 1980 and 2020. Part 2.

Risk Factor	First Author * [Ref.]	Year	Country	Design	Representativeness	Exposed	Cases	Controls	Age †
CIN (n.o.s.)	Jakobsson [55]	2011	Finland	C	Nationwide, population-based cohort	26,876	12	NA	75% <40
CIN1-3	Kalliala [56]	2005	Finland	C	Single-centre hospital-based cohort	7564	6	NA	Mean, 34
CIN2-3	Gaudet [47]	2014	Canada	C	Cohort of attenders to a population-based CSP	54,32	96	NA	Mean, 35
CIN2-3	Preti [71]	2020	Italy	C	Single-hospital-based cohort	3184	1	NA	NR
CIN3	Bjørge [25]	1995	Norway	C	Nationwide, population-based cohort	37,001	32	NA	74% <40
CIN3	Evans [42]	2003	UK	C	Regional, population-based cohort	59,519	24	NA	74% <40
CIN3	Edgren [40]	2007	Sweden	C	Nationwide, population-based cohort	125,292	94	NA	Mean, 35
CIN3	Coffey [33]	2016	UK	C	Cohort of attenders to a population-based MSP	1,300,042	898	NA	Range, 49–65
CIN3	Ebisch [39]	2017	The Neth.	C	Nationwide, population-based cohort	89,018	129	NA	Median, 35
CIN3	Pan [67]	2019	UK	C	Regional, population-based cohort	69,714	62	NA	Median, 30
VLS	Halonen [48]	2017	Finland	C	Nationwide, population-based cohort	7616	182	NA	78% ≥50
VLS	Corazza [34]	2019	Italy	C	Provincial, population-based cohort	308	7	NA	NR
SLE	Mellemkjaer [62]	1997	Denmark	C	Nationwide, population-based cohort	1308	3	NA	71% <60
SLE	Parikh-Patel [69]	2008	US	C	Statewide, population-based cohort	27,133	49	NA	NR
SLE	Chen [31]	2010	Taiwan	C	Nationwide, population-based cohort	10,394	3	NA	NR
SLE	Dreyer [38]	2011	Denmark	C	8-hospital-based cohort	NR	2	NA	NR
SLE	Bernatsky [22]	2013	Canada	C	Multicentre, international hospital cohort	14,768	7	NA	NR
Rheumatoid arthritis	Parikh-Patel [70]	2009	US	C	Statewide, population-based cohort	65,236	56	NA	NR
Rheumatoid arthritis	Chen [32]	2011	Taiwan	C	Nationwide, population-based cohort	18,527	5	NA	NR
Age at menarche	Mabuchi [58]	1985	US	CC	Multicentre hospital-based case series	NA	149	149	81% ≥50
Age at menarche	Coffey [33]	2016	UK	C	Cohort of attenders to a population-based MSP	1,300,042	877	NA	Range, 49–65
Age at menarche	Brinton [30]	2017	US	CC	8-state/area cohort of registered retired persons	201,469	170	NA	Mean, 61
Pregnancy	Sherman [79]	1994	US	CC	3-county, cancer-registry-based case series	NA	81	1010	Mean, 59
Age at first pregnancy	Mabuchi [58]	1985	US	CC	Multicentre hospital-based case series	NA	149	149	81% ≥50
Age at first pregnancy	Sherman [79]	1994	US	CC	3-county, cancer-registry-based case series	NA	81	1010	Mean, 59
No. of pregnancies	Mabuchi [58]	1985	US	CC	Multicentre hospital-based case series	NA	149	149	81% ≥50
No. of pregnancies	Sherman [79]	1994	US	CC	3-county, cancer-registry-based case series	NA	81	1010	Mean, 59
Parity	Sherman [79]	1994	US	CC	3-county, cancer-registry-based case series	NA	81	1010	Mean, 59
Parity	Parazzini [68]	1995	Italy	CC	Hospital-based case series	NA	125	541	Median, 63
Parity	Coffey [33]	2016	UK	C	Cohort of attenders to a population-based MSP	1,300,042	897	NA	Range, 49–65
Age at first live birth	Sherman [79]	1994	US	CC	3-county, cancer-registry-based case series	NA	81	1010	Mean, 59
Age at first birth	Brinton [30]	2017	US	CC	8-state/area cohort of registered retired persons	201,469	170	NA	Mean, 61
No. of live births	Sherman [79]	1994	US	CC	3-county, cancer-registry-based case series	NA	81	1010	Mean, 59
No. of births	Brinton [30]	2017	US	CC	8-state/area cohort of registered retired persons	201,469	170	NA	Mean, 61
Menopausal status	Mabuchi [58]	1985	US	CC	Multicentre hospital-based case series	NA	149	149	81% ≥50
Menopausal status	Parazzini [68]	1995	Italy	CC	Hospital-based case series	NA	125	541	Median, 63
Age at menopause	Mabuchi [58]	1985	US	CC	Multicentre hospital-based case series	NA	149	149	81% ≥50
Age at menopause	Coffey [33]	2016	UK	C	Cohort of attenders to a population-based MSP	412,633	325	NA	Range, 49–65
Age at menopause	Brinton [30]	2017	US	C	8-state/area cohort of registered retired persons	201,469	170	NA	Mean, 61
**Risk Factor**	**[Ref.]**	**Comparison**	**Disease**	**Measure**	**Result (95% CI)**	**Adjustment variables**			
CIN (n.o.s.)	[55]	Exposed vs. general population	VC	SIR	6.15 (3.18–10.7)	Age, period			
CIN1-3	[56]	Exposed vs. general population	VC	SIR	4.1 (1.5–8.9)	Age, period			
CIN2-3	[47]	Exposed vs. general population	VC	SIR	2.90 (1.71–4.61)	Age			
CIN2-3	[71]	Exposed vs. general population	VC	SIR	1.70 (0.04–9.59)	Age, period, municipality			
CIN3	[25]	Exposed vs. general population	V/VC	SIR	4.04 (2.76–5.70)	Age			
CIN3	[42]	Exposed vs. general population	VC	SIR	4.4 (2.8–6.6)	Age, period			
CIN3	[40]	Exposed vs. general population	VC	IRR	2.22 (1.79–2.73)	Age, period, SES, parity			
CIN3	[33]	Exposed vs. general population	VC	RR	2.68 (1.71–4.18)	Age, DEPRI, smoking, alcohol, BMI, D, age at M, parity, OC use, HYST			
CIN3	[39]	Exposed vs. a general population sample	VC	IRR	4.97 (3.26–7.57)	Age, follow-up period			
CIN3	[67]	Exposed vs. general population	VC	SIR	2.8 (2.2–3.6)	Age, year			
VLS	[48]	Exposed vs. general population	VSCC	SIR	33.6 (28.9–38.6)	Age, period, follow-up period			
VLS	[34]	Exposed vs. general population	VC	SIR	39.58 (15.91–81.54)	Age			
SLE	[62]	Exposed vs. general population	V/VC	SIR	5.7 (1.2–16.6)	Age, period			
SLE	[69]	Exposed vs. general population	V/VC	SIR	3.27 (2.41–4.31)	Age, race/ethnicity			
SLE	[31]	Exposed vs. general population	V/VC	SIR	4.76 (4.24–5.33)	Age, period			
SLE	[38]	Exposed vs. general population	V/VC	SIR	9.1 (2.3–36.5)	Age, period			
SLE	[22]	Exposed vs. general population	VC	SIR	3.78 (1.52–7.78)	Age, year			
Rheumatoid arthritis	[70]	Exposed vs. general population	V/VC	SIR	0.99 (0.75–1.29)	Age, race/ethnicity			
Rheumatoid arthritis	[32]	Exposed vs. general population	V/VC	SIR	1.69 (1.54–1.84)	Age, period			
Age at menarche	[58]	≥16 vs. <12	VC	OR	1.43 (NS)	NR			
Age at menarche	[33]	≥14 vs. <14	VC	RR	1.04 (0.90–1.19)	Age, DEPRI, smoking, alcohol, BMI, D, parity, OC use, HYST, CIN3			
Age at menarche	[30]	≥15 vs. ≤12	VC	HR	1.27 (0.75–2.15)	Age, race, smoking, BMI, marital status, OC use, menopausal hormone			
Pregnancy	[79]	No vs. yes	VSCC	OR	0.8 (0.4–1.9)	Age, EDU, smoking, no. of sexual partners, genital warts			
Age at first pregnancy	[58]	≥35 vs. <20	VC	OR	2.00 (NS)	NR			
Age at first pregnancy	[79]	≥25 vs. <20	VSCC	OR	1.0 (0.4–2.1)	Age, EDU, smoking, no. of sexual partners, genital warts			
No. of pregnancies	[58]	≥3 vs. 0	VC	OR	0.65 (NS)	NR			
No. of pregnancies	[79]	≥3 vs. 0	VSCC	OR	1.2 (0.5–2.9)	Age, EDU, smoking, no. of sexual partners, genital warts			
Parity	[79]	Nulliparous vs. multiparous	VSCC	OR	1.3 (0.7–2.4)	Age, EDU, smoking, no. of sexual partners, genital warts			
Parity	[68]	≥3 vs. 0	VC	OR	0.8 (0.4–1.5)	Age, EDU, BMI			
Parity	[33]	Nulliparous vs. parous	VC	RR	1.19 (0.97–1.47)	Age, DEPRI, smoking, alcohol, BMI, D, age at M, OC, HYST, CIN3			
Age at first live birth	[79]	≥25 vs. <20	VSCC	OR	0.8 (0.4–1.9)	Age, EDU, smoking, no. of sexual partners, genital warts			
Age at first birth	[30]	≥30 vs. <20	VC	HR	0.83 (0.35–1.92)	Age, race, smoking, BMI, marital status, OC, menopausal hormone			
No. of live births	[79]	≥3 vs. 0	VSCC	OR	0.9 (0.5–1.8)	Age, EDU, smoking, no. of sexual partners, genital warts			
No. of births	[30]	≥5 vs. 0	VC	HR	1.22 (0.60–2.46)	Age, race, smoking, BMI, marital status, OC, menopausal hormone			
Menopausal status	[58]	Post- vs. premenopausal	VC	OR	1.15 (NS)	NR			
Menopausal status	[68]	Post- vs. pre-/perimenopause	VC	OR	0.4 (0.2–1.1)	Age, EDU, BMI			
Age at menopause	[58]	≥50 vs. <35	VC	OR	0.86 (NS)	NR			
Age at menopause	[33]	<50 vs. ≥50	VSCC	RR	1.59 (1.22–1.89)	DEPRI, smoking, alcohol, BMI, D, age at M, parity, OC, HYST, CIN3			
Age at menopause	[30]	<45 vs. 50–54	VC	HR	0.74 (0.35–1.58)	Age, race, smoking, BMI, marital status, OC, menopausal hormone			

BMI = body mass index; C = cohort; CC = case-control; CIN = cervical intraepithelial neoplasia; CSP = cervical screening programme; D = diabetes; DEPRI = deprivation; EDU = education; HR = hazard ratio; HYST = hysterectomy; IRR = incidence rate ratio; M = menarche; MSP = mammography screening programme; n.o.s. = not otherwise specified; NA = not applicable; Neth. = Netherlands; NR = not reported; NS = not significant; OC = oral contraceptive; OR = odds ratio; ref. = reference; RR = relative risk; SES = socioeconomic status; SIR = standardised incidence ratio; SLE = systemic lupus erythematosus; UK = United Kingdom; US = United States; V/VC = vulvar/vaginal cancer; VC = vulvar cancer; VLS = vulvar lichen sclerosus; vs. = versus; VSCC = vulvar squamous cell carcinoma. * In the case of international authorship, the country of the first author is indicated. † The best information made available in the article is indicated. In general, age refers to the age of cases at diagnosis in case-control studies and the age at entry into cohort studies. If possible, age is expressed in completed years.

**Table 4:** Summary of cohort and case-control studies on all known and putative risk factors for vulvar cancer published between 1980 and 2020. Part 3.

Risk Factor	First Author * [Ref.]	Year	Country	Design	Representativeness	Exposed	Cases	Controls	Age †
Induced abortion	Sherman [79]	1994	US	CC	3-county, cancer-registry-based case series	NA	81	1010	Mean, 59
Miscarriage	Sherman [79]	1994	US	CC	3-county, cancer-registry-based case series	NA	81	1010	Mean, 59
Prior tubal ligation	Coffey [33]	2016	UK	C	Cohort of attenders to a population-based MSP	1,300,042	878	NA	Range, 49–65
Prior hysterectomy	Coffey [33]	2016	UK	C	Cohort of attenders to a population-based MSP	1,300,042	718	NA	Range, 49–65
Prior hysterectomy	Brinton [30]	2017	US	CC	8-state/area cohort of registered retired persons	201,469	170	NA	Mean, 61
OC use	Sherman [79]	1994	US	CC	3-county, cancer-registry-based case series	NA	81	1010	Mean, 59
OC use	Coffey [33]	2016	UK	C	Cohort of attenders to a population-based MSP	1,300,042	884	NA	Range, 49–65
OC use	Brinton [30]	2017	US	CC	8-state/area cohort of registered retired persons	201,469	170	NA	Mean, 61
Oestrogen use	Sherman [79]	1994	US	CC	3-county, cancer-registry-based case series	NA	81	1010	Mean, 59
Menopausal hormone use	Coffey [33]	2016	UK	C	Cohort of attenders to a population-based MSP	917,711	653	NA	Range, 49–65
Menopausal hormone use	Brinton [30]	2017	US	CC	8-state/area cohort of registered retired persons	201,469	170	NA	Mean, 61
Metabolic syndrome	Nagel [64]	2011	Germany	C	3-country cohort from primary prevention programmes	288,834	82	NA	Mean, 44
Blood glucose	Nagel [64]	2011	Germany	C	3-country cohort from primary prevention programmes	288,834	82	NA	Mean, 44
Triglyceride concentration	Nagel [64]	2011	Germany	C	3-country cohort from primary prevention programmes	288,834	82	NA	Mean, 44
Cholesterol concentration	Nagel [64]	2011	Germany	C	3-country cohort from primary prevention programmes	288,834	82	NA	Mean, 44
Diabetes	Coffey [33]	2016	UK	C	Cohort of attenders to a population-based MSP	1,300,042	897	NA	Range, 49–65
Diabetes	Brinton [30]	2017	US	CC	8-state/area cohort of registered retired persons	201,469	170	NA	Mean, 61
BMI	Sherman [79]	1994	US	CC	3-county, cancer-registry-based case series	NA	81	1010	Mean, 59
BMI	Parazzini [68]	1995	Italy	CC	Hospital-based case series	NA	125	541	Median, 63
BMI	Parazzini [68]	1995	Italy	CC	Hospital-based case series	NA	125	541	Median, 63
BMI	Parazzini [68]	1995	Italy	CC	Hospital-based case series	NA	125	541	Median, 63
BMI	Nagel [64]	2011	Germany	C	3-country cohort from primary prevention programmes	288,834	82	NA	Mean, 44
BMI	Coffey [33]	2016	UK	C	Cohort of attenders to a population-based MSP	1,300,042	638	NA	Range, 49–65
BMI	Coffey [33]	2016	UK	C	Cohort of attenders to a population-based MSP	1,300,042	545	NA	Range, 49–65
BMI	Brinton [30]	2017	US	CC	8-state/area cohort of registered retired persons	201,469	170	NA	Mean, 61
Coffee consumption	Mabuchi [58]	1985	US	CC	Multicentre hospital-based case series	NA	149	149	81% ≥50
Coffee consumption	Mabuchi [58]	1985	US	CC	Multicentre hospital-based case series	NA	149	149	81% ≥50
Coffee consumption	Parazzini [68]	1995	Italy	CC	Hospital-based case series	NA	125	541	Median, 63
Meat consumption	Parazzini [68]	1995	Italy	CC	Hospital-based case series	NA	125	541	Median, 63
Meat consumption	Parazzini [68]	1995	Italy	CC	Hospital-based case series	NA	125	541	Median, 63
Green vegetable consumption	Parazzini [68]	1995	Italy	CC	Hospital-based case series	NA	125	541	Median, 63
Green vegetable consumption	Parazzini [68]	1995	Italy	CC	Hospital-based case series	NA	125	541	Median, 63
Carrot consumption	Parazzini [68]	1995	Italy	CC	Hospital-based case series	NA	125	541	Median, 63
Carrot consumption	Parazzini [68]	1995	Italy	CC	Hospital-based case series	NA	125	541	Median, 63
Alcohol consumption	Parazzini [68]	1995	Italy	CC	Hospital-based case series	NA	125	541	Median, 63
Alcohol consumption	Weiderpass [87]	2001	Sweden	C	Nationwide, population-based cohort	36,856	8	NA	Mean, 42
Alcohol consumption	Madsen [61]	2008	Denmark	CC	Nationwide, cancer-registry-based series	NA	116	518	Median, 63
Alcohol consumption	Coffey [33]	2016	UK	C	Cohort of attenders to a population-based MSP	1,300,042	890	NA	Range, 49–65
Alcohol consumption	Brinton [30]	2017	US	CC	8-state/area cohort of registered retired persons	201,469	170	NA	Mean, 61
**Risk factor**	**[Ref.]**	**Comparison**	**Disease**	**Measure**	**Result (95% CI)**	**Adjustment variables**			
Induced abortion	[79]	Exposure vs. no exposure	VSCC	OR	1.9 (1.0–3.8)	Age, EDU, smoking, no. of sexual partners, genital warts			
Miscarriage	[79]	Exposure vs. no exposure	VSCC	OR	0.9 (0.5–1.7)	Age, EDU, smoking, no. of sexual partners, genital warts			
Prior tubal ligation	[33]	Exposure vs. no exposure	VC	RR	0.91 (0.77–1.07)	Age, DEPRI, smoking, alcohol, BMI, D, age at M, parity, OC, HYST, CIN3			
Prior hysterectomy	[33]	HYST + oophorect. vs. no HYST	VC	RR	1.08 (0.83–1.39)	Age, DEPRI, smoking, alcohol, BMI, D, age at M, parity, OC, CIN3			
Prior hysterectomy	[30]	Exposure vs. no exposure	VC	HR	1.30 (0.92–1.83)	Age, race, smoking, BMI, marital status, OC, menopausal hormone			
OC use	[79]	≥5 years vs. never	VSCC	OR	0.4 (0.2–1.3)	Age, EDU, smoking, no. of sexual partners, genital warts			
OC use	[33]	Ever vs. never	VC	RR	1.08 (0.94–1.24)	Age, DEPRI, smoking, alcohol, BMI, D, age at M, parity, HYST, CIN3			
OC use	[30]	≥10 years vs. <1	VC	HR	0.75 (0.39–1.45)	Age, race, smoking, BMI, marital status, menopausal hormone			
Oestrogen use	[79]	Ever vs. never	VSCC	OR	1.2 (0.6–2.3)	Age, EDU, smoking, no. of sexual partners, genital warts			
Menopausal hormone use	[33]	Current vs. never	VC	RR	0.86 (0.73–1.02)	Age, DEPRI, smoking, alcohol, BMI, D, age at M, parity, OC, HYST, CIN3			
Menopausal hormone use	[30]	Current, ≥10 years vs. never	VC	HR	0.88 (0.58–1.36)	Age, race, smoking, BMI, marital status, OC			
Metabolic syndrome	[64]	For 1 SD INC in the stand. z-score	VC	HR	1.78 (1.30–2.41)	Age, smoking			
Blood glucose	[64]	For 1 SD INC in the stand. z-score	VC	HR	1.98 (1.10–3.58)	Age, smoking			
Triglyceride concentration	[64]	For 1 SD INC in the stand. z-score	VC	HR	2.09 (1.39–3.15)	Age, smoking			
Cholesterol concentration	[64]	For 1 SD INC in the stand. z-score	VC	HR	1.08 (0.77–1.49)	Age, smoking			
Diabetes	[33]	Exposure vs. no exposure	VC	RR	0.87 (0.58–1.30)	Age, DEPRI, smoking, alcohol, BMI, age at M, parity, OC, HYST, CIN3			
Diabetes	[30]	Exposure vs. no exposure	VC	HR	1.05 (0.58–1.93)	Age, race, smoking, BMI, marital status, OC, menopausal hormone			
BMI	[79]	Highest vs. lowest category	VSCC	OR	2.9 (1.5–5.8)	Age, EDU, smoking, no. of sexual partners, genital warts			
BMI	[68]	23.5–25.3 vs. <21.3	VC	OR	1.8 (0.8–3.6)	Age, EDU			
BMI	[68]	25.4–28.1 vs. <21.3	VC	OR	2.5 (1.2–5.0)	Age, EDU			
BMI	[68]	≥28.2 vs. 21.3	VC	OR	2.5 (1.2–5.2)	Age, EDU			
BMI	[64]	For 1 SD INC in the stand. z-score	VC	HR	1.36 (1.11–1.69)	Age, smoking			
BMI	[33]	25.0–29.9 vs. <25.0	VC	RR	1.19 (1.02–1.39)	Age, DEPRI, smoking, alcohol, D, age at M, parity, OC, HYST, CIN3			
BMI	[33]	≥30.0 vs. <25.0	VC	RR	1.71 (1.44–2.04)	Age, DEPRI, smoking, alcohol, D, age at M, parity, OC, HYST, CIN3			
BMI	[30]	≥30.0 vs. <25.0	VC	HR	1.62 (1.10–2.40)	Age, race, smoking, marital status, OC, menopausal hormone			
Coffee consumption	[58]	3–4 cups/day vs. <1	VC	OR	2.99 (NR, *p* < 0.05)	NR			
Coffee consumption	[58]	≥5 cups/day vs. <1	VC	OR	2.42 (NR, *p* < 0.05)	NR			
Coffee consumption	[68]	≥3 cups/day vs. 0	VC	OR	0.8 (0.4–1.3)	Age, EDU, BMI			
Meat consumption	[68]	4–5 portions/week vs. ≥6 portions	VC	OR	1.0 (0.5–1.8)	Age, EDU, BMI			
Meat consumption	[68]	<4 portions/week vs. ≥6	VC	OR	1.5 (0.9–2.4)	Age, EDU, BMI			
Green vegetable consumption	[68]	7–13 portions/week vs. ≥14	VC	OR	1.1 (0.6–1.8)	Age, EDU, BMI			
Green vegetable consumption	[68]	<7 portions/week vs. ≥14	VC	OR	2.0 (1.2–3.4)	Age, EDU, BMI			
Carrot consumption	[68]	1 portion/week vs. ≥2	VC	OR	1.3 (0.7–2.2)	Age, EDU, BMI			
Carrot consumption	[68]	<1 portion/week vs. ≥2	VC	OR	1.4 (0.9–2.2)	Age, EDU, BMI			
Alcohol consumption	[68]	Regular vs. never	VC	OR	1.1 (0.7–1.7)	Age, EDU, BMI			
Alcohol consumption	[87]	Exposed vs. general population	VSCC	SIR	1.0 (0.4–2.0)	Age, year			
Alcohol consumption	[61]	0 consumption-years vs. <10	VSCC	OR	0.37 (0.20–0.70)	Age, EDU, smoking, marital status, anogenital warts			
Alcohol consumption	[33]	≥3 units/week vs. 0–2	VC	RR	0.87 (0.75–1.00)	Age, DEPRI, smoking, BMI, D, age at M, parity, OC, HYST, CIN3			
Alcohol consumption	[30]	≥1.0 vs. 0	VC	HR	0.77 (0.44–1.33)	Age, race, smoking, BMI, marital status, OC, menopausal hormone			

BMI = body mass index; C = cohort; CC = case-control; CIN = cervical intraepithelial neoplasia; D = diabetes; DEPRI = deprivation; EDU = education; HR = hazard ratio; HYST = hysterectomy; INC = increment; M = menarche; MSP = mammography screening programme; NA = not applicable; NR = not reported; OC = oral contraceptive; oophorect. = oophorectomy; OR = odds ratio; ref. = reference; RR = relative risk; SD = standard deviation; stand. = standardised; UK = United Kingdom; US = United States; VC = vulvar cancer; vs. = versus; VSCC = vulvar squamous cell carcinoma. * In the case of international authorship, the country of the first author is indicated. † The best information made available in the article is indicated. In general, age refers to the age of cases at diagnosis in case-control studies and the age at entry into cohort studies. If possible, age is expressed in completed years.

**Table 5:** Summary of cohort and case-control studies on all known and putative risk factors for vulvar cancer published between 1980 and 2020. Part 4.

Risk Factor	First Author * [Ref.]	Year	Country	Design	Representativeness	Exposed	Cases	Controls	Age †
Smoking	Mabuchi [58]	1985	US	CC	Multicentre hospital-based case series	NA	149	149	81% ≥50
Smoking	Brinton [29]	1990	US	CC	Multicentre hospital-based case series	NA	209	113	Mean, 54
Smoking	Daling [36]	1992	US	CC	13-county, cancer-registry-based case series	NA	295	902	69% <60
Smoking	Daling [36]	1992	US	CC	13-county, cancer-registry-based case series	NA	295	902	69% <60
Smoking	Daling [36]	1992	US	CC	13-county, cancer-registry-based case series	NA	295	902	69% <60
Smoking	Daling [36]	1992	US	CC	13-county, cancer-registry-based case series	NA	295	902	69% <60
Smoking	Daling [36]	1992	US	CC	13-county, cancer-registry-based case series	NA	295	902	69% <60
Smoking	Daling [36]	1992	US	CC	13-county, cancer-registry-based case series	NA	295	902	69% <60
Smoking	Parazzini [68]	1995	Italy	CC	Hospital-based case series	NA	125	541	Median, 63
Smoking	Madeleine [59]	1997	US	CC	3-county, cancer-registry-based case series	NA	110	1403	52% ≥60
Smoking	Madeleine [59]	1997	US	CC	3-county, cancer-registry-based case series	NA	110	1403	52% ≥60
Smoking	Madeleine [59]	1997	US	CC	3-county, cancer-registry-based case series	NA	110	1403	52% ≥60
Smoking	Madsen [61]	2008	Denmark	CC	Nationwide, cancer-registry-based series	NA	116	518	Median, 63
Smoking	Coffey [33]	2016	UK	C	Cohort of attenders to a population-based MSP	1,300,042	624	NA	Range, 49–65
Smoking	Brinton [30]	2017	US	CC	8-state/area cohort of registered retired persons	201,469	170	NA	Mean, 61
HIV	Silverberg [80]	2009	US	C	Healthcare delivery system cohort	NR	12	NA	≥18
HIV	Franzetti [44]	2013	Italy	C	Single-hospital-based cohort	1542	5	NA	Median, 42
HIV	Hernández-Ramírez [50]	2017	US	C	Multistate, population-based cohort	NR	151	NA	NR
HIV	Mpunga [63]	2018	Rwanda	CC	Hospital-based case series	NA	23	960	NR
HIV	Ortiz [66]	2018	US	C	Multistate, population-based cohort	NR	28	NA	NR
HIV-AIDS	Hessol [51]	2018	US	C	Metropolitan, population-based cohort	1338	14	NA	≥16 years
AIDS	Frisch [46]	2000	US	C	Multistate, population-based cohort	51,760	12	NA	Median, 33
AIDS	Tanaka [83]	2018	Brazil	C	Metropolitan, population-based cohort	NR	14	NA	≥13
Dialysis	Fairley [43]	1994	Australia	C	2-nationwide, population-based cohort	NR	2	NA	≥15
Dialysis/renal transplantation	Skov Dalgaard [82]	2013	Denmark	C	Nationwide, population-based cohort	4610	15	NA	≥14
Renal transplantation	Fairley [43]	1994	Australia	C	2-nationwide, population-based cohort	NR	24	NA	≥15
Renal transplantation	Birkeland [23]	1995	Denmark	C	4-nation, population-based cohort	2369	11	NA	NR
Renal transplantation	Vajdic [84]	2006	Australia	C	2-nationwide, population-based cohort	12,485	18	NA	Mean, 50
Renal transplantation	Villeneuve [86]	2007	Canada	C	Nationwide, population-based cohort	4100	3	NA	NR
Renal transplantation	Reinholdt [73]	2020	Denmark	C	Nationwide, population-based cohort	1588	8	NA	63% ≥40
S-O transplantation	Adami [21]	2003	Sweden	C	Nationwide, population-based cohort	2339	9	NA	NR
S-O transplantation	Engels [41]	2011	US	C	Multistate, population-based cohort	68,705	58	NA	NR
S-O transplantation	Madeleine [60]	2013	US	C	Nationwide, population-based cohort	72,035	66	NA	≥18
Paediatric S-O transplantation	Simard [81]	2011	Sweden	C	Nationwide, population-based cohort	NR	3	NA	<18
Paediatric S-O transplantation	Yanik [88]	2017	US	C	Multistate, population-based cohort	8210	2	NA	<18
Liver transplantation	Schrem [77]	2013	Germany	C	Single-hospital-based cohort	940	5	NA	NR
**Risk factor**	**[Ref.]**	**Comparison**	**Disease**	**Measure**	**Result (95% CI)**	**Adjustment variables**			
Smoking	[58]	10–20 cig./day vs. 0	VC	OR	2.46 (NR, *p* < 0.05)	NR			
Smoking	[29]	Current smoker vs. never	VC	RR	1.19 (0.6–2.2)	Age, no. of sexual partners, genital warts, previous abnormal Pap smear			
Smoking	[36]	Current vs. never	VSCC	OR	4.8 (3.3–6.8)	Age, geographic location, no. of sexual partners			
Smoking	[36]	Former vs. never	VSCC	OR	1.8 (1.2–2.8)	Age, geographic location, no. of sexual partners			
Smoking	[36]	<20 cig./day (current) vs. 0	VSCC	OR	3.3 (2.0–5.3)	Age, geographic location, no. of sexual partners			
Smoking	[36]	≥40 cig./day (current) vs. 0	VSCC	OR	6.6 (3.5–12.3)	Age, geographic location, no. of sexual partners			
Smoking	[36]	Age started <17 (current) vs. none	VSCC	OR	6.8 (4.4–10.6)	Age, geographic location, no. of sexual partners			
Smoking	[36]	Age started ≥20 (current) vs. none	VSCC	OR	3.3 (2.0–5.5)	Age, geographic location, no. of sexual partners			
Smoking	[68]	Ever vs. never	VC	OR	1.1 (0.7–1.8)	Age, EDU, BMI			
Smoking	[59]	Ever vs. never	VSCC	OR	2.2 (1.3–3.7)	Age, EDU, BMI, HPV 16 seropositivity			
Smoking	[59]	Former vs. never	VSCC	OR	1.4 (0.7–2.8)	Age, EDU, BMI, HPV 16 seropositivity			
Smoking	[59]	Current vs. never	VSCC	OR	3.0 (1.7–5.3)	Age, EDU, BMI, HPV 16 seropositivity			
Smoking	[61]	Current vs. never	VSCC	OR	2.61 (1.53–4.46)	Age, EDU, alcohol, marital status, anogenital warts			
Smoking	[33]	Current vs. never	VC	RR	1.04 (0.87–1.26)	Age, DEPRI, alcohol, BMI, D, age at M, parity, OC, HYST, CIN3			
Smoking	[30]	Current vs. never	VC	HR	1.86 (1.21–2.87)	Age, race, BMI, marital status, OC, menopausal hormone			
HIV	[80]	Exposed vs. unexposed	V/VC	RR	19.5 (9.2–41.1)	Age, year, race/ethnicity			
HIV	[44]	Exposed vs. general population	VC	SIR	69.2 (22.3–161.4)	Age			
HIV	[50]	Exposed vs. general population	VC	SIR	9.35 (7.91–10.96)	Age, year, race/ethnicity, registry			
HIV	[63]	Cases vs. hospital controls	VC	OR	17.8 (6.3–50.1)	Age, place of residence			
HIV	[66]	Exposed vs. general Hispanic population	VC	SIR	9.03 (6.00–13.1)	Age, year, registry			
HIV-AIDS	[51]	Exposed vs. general population	VC	SIR	13.3 (6.1–20.6)	Age, year, race			
AIDS	[46]	Exposed vs. general population	V/VSCC	RR	5.8 (3.0–10.2)	Age, race			
AIDS	[83]	Exposed vs. general population	V/VC	SIR	6.78 (4.02–11.45)	Age			
Dialysis	[43]	Exposed vs. general population	VC	SIR	4.2 (0.4–11.9)	Age			
Dialysis/renal transplantation	[82]	Exposed vs. a population control cohort	V/VC	IRR	5.81 (3.36–10.1)	Age, comorbidity			
Renal transplantation	[43]	Exposed vs. general population	VC	SIR	55.8 (35.8–83.0)	Age			
Renal transplantation	[23]	Exposed vs. general population	V/VC	SIR	31.0 (15.0–55.0)	Age, period			
Renal transplantation	[84]	Exposed vs. general population	VC	SIR	24.7 (S)	Age			
Renal transplantation	[86]	Exposed vs. general population	VC	SIR	5.5 (1.1–16.0)	Age, year			
Renal transplantation	[73]	Exposed vs. a cohort of unaffected controls	VSCC	HR	31.0 (13.3–72.0)	Age, EDU, income			
S-O transplantation	[21]	Exposed vs. general population	VC	SIR	26.2 (12.0–49.8)	Age, year			
S-O transplantation	[41]	Exposed vs. general population	VC	SIR	7.60 (5.77–9.83)	Age, year, race/ethnicity, registry			
S-O transplantation	[60]	Exposed vs. general population	VC	SIR	7.3 (5.6–9.2)	Age, year, race/ethnicity			
Paediatric S-O transplantation	[81]	Exposed vs. general population	V/VC	SIR	665.0 (137.1–1934.4)	Age, year			
Paediatric S-O transplantation	[88]	Exposed vs. general population	VC	SIR	17.4 (S)	Age, year, race/ethnicity			
Liver transplantation.	[77]	Exposed vs. general population	VC	SIR	23.80 (7.70–55.50)	Age			

AIDS = acquired immunodeficiency syndrome; BMI = body mass index; C = cohort; CC = case-control; CIN = cervical intraepithelial neoplasia; D = diabetes; DEPRI = deprivation; EDU = education; HIV = human immunodeficiency virus; HPV = human papillomavirus; HR = hazard ratio; HYST = hysterectomy; IRR = incidence rate ratio; M = menarche; MSP = mammography screening programme; NA = not applicable; NR = not reported; OC = oral contraceptive; OR = odds ratio; ref. = reference; RR = relative risk; S = significant; S-O = solid-organ; SIR = standardised incidence ratio; UK = United Kingdom; US = United States; V/VC = vulvar/vaginal cancer; V/VSCC = vulvar/vaginal squamous cell carcinoma; VC = vulvar cancer; vs. = versus; VSCC = vulvar squamous cell carcinoma. * In the case of international authorship, the country of the first author is indicated. † The best information made available in the article is indicated. In general, age refers to the age of cases at diagnosis in case-control studies and the age at entry into cohort studies. If possible, age is expressed in completed years.

**Table 6:** Summary of cohort and case-control studies on all known and putative risk factors for vulvar cancer published between 1980 and 2020. Part 5.

Risk Factor	First Author * [Ref.]	Year	Country	Design	Representativeness	Exposed	Cases	Controls	Age †
Breast implants	Brinton [28]	2001	US	C	Multicentre hospital-based cohort	13,488	10	NA	Mean, 34
Breast implants	Deapen [37]	2007	US	C	Multicentre hospital-based cohort	3139	2	NA	NR
Fanconi anaemia	Rosenberg [75]	2003	US	C	Cohort of patients known to a research fund	69	3	NA	Median, 4
Fanconi anaemia	Rosenberg [74]	2008	US	C	Cohort collected through professional contacts	78	3	NA	NR
Previous abnormal Pap smear	Brinton [29]	1990	US	CC	Multicentre hospital-based case series	NA	209	111	Median, 54
Previous abnormal Pap smear	Sherman [78]	1991	US	CC	3-county, cancer-registry-based case series	NA	53	466	Range, 18–79
Previous abnormal Pap smear	Viikki [85]	1998	Finland	C	Nationwide, population-based screening cohort	4095	7	NA	Range, 30–60
Education	Parazzini [68]	1995	Italy	CC	Hospital-based case series	NA	125	541	Median, 63
Education	Madsen [61]	2008	Denmark	CC	Nationwide, cancer-registry-based series	NA	116	518	Median, 63
**Risk factor**	**[Ref.]**	**Comparison**	**Disease**	**Measure**	**Result (95% CI)**	**Adjustment variables**			
Breast implants	[28]	Exposed vs. general population	V/VC	SIR	2.51 (1.1–5.6)	Age, calendar year, race			
Breast implants	[37]	Exposed vs. general population	VC	SIR	4.40 (0.48–15.89)	Age, period			
Fanconi anaemia	[75]	Exposed vs. general population	VC	SIR	4317 (870–12,615)	Age, birth cohort			
Fanconi anaemia	[74]	Exposed vs. general population	VC	SIR	2411 (S)	Age			
Previous abnormal Pap smear	[29]	Exposure vs. no exposure	VC	RR	1.41 (0.5–3.6)	Age, smoking, no. of sexual partners, genital warts			
Previous abnormal Pap smear	[78]	Exposure vs. no exposure	VSCC	OR	5.0 (2.3–10.7)	Age			
Previous abnormal Pap smear	[85]	Exposed vs. general population	VC	SIR	5.8 (2.3–12.0)	Age, period, follow-up period			
Education	[68]	12 years vs. <7	VC	OR	0.5 (0.3–1.2)	Age, BMI			
Education	[61]	≥10 years vs. <10	VSCC	OR	0.53 (0.31–0.90)	Age, smoking, alcohol, marital status, anogenital warts			

BMI = body mass index; C = cohort; CC = case-control; NA = not applicable; NR = not reported; OR = odds ratio; ref. = reference; RR = relative risk; S = significant; SIR = standardised incidence ratio; US = United States; V/VC = vulvar/vaginal cancer; VC = vulvar cancer; vs. = versus; VSCC = vulvar squamous cell carcinoma. * In the case of international authorship, the country of the first author is indicated. † The best information made available in the article is indicated. In general, age refers to the age of cases at diagnosis in case-control studies and the age at entry into cohort studies. If possible, age is expressed in completed years.

## Data Availability

All authors had full access to all the data in the study and take responsibility for the integrity of data and the accuracy of data analysis. The data are available from the corresponding author upon reasonable request.
